# Glial Cell Line–Derived Neurotrophic Factor Receptor Rearranged During Transfection Agonist Supports Dopamine Neurons *In Vitro* and Enhances Dopamine Release *In Vivo*

**DOI:** 10.1002/mds.27943

**Published:** 2019-12-16

**Authors:** Arun Kumar Mahato, Jaakko Kopra, Juho‐Matti Renko, Tanel Visnapuu, Ilari Korhonen, Nita Pulkkinen, Maxim M. Bespalov, Andrii Domanskyi, Eric Ronken, T. Petteri Piepponen, Merja H. Voutilainen, Raimo K. Tuominen, Mati Karelson, Yulia A. Sidorova, Mart Saarma

**Affiliations:** ^1^ Laboratory of Molecular Neuroscience, Institute of Biotechnology, Helsinki Institute of Life Science, Viikinkaari 5D University of Helsinki Helsinki Finland; ^2^ Division of Pharmacology and Pharmacotherapy, Faculty of Pharmacy, Viikinkaari 5E University of Helsinki Helsinki Finland; ^3^ Solvay Pharmaceuticals Weesp Netherlands; ^4^ Institute of Chemistry Tartu University Tartu Estonia

**Keywords:** Clustered Regularly Interspaced Short Palindromic Repeats/CRISPR associated protein 9, dopamine neurons, glial cell line‐derived neurotrophic factor (GDNF), Parkinson's disease, receptor tyrosine kinase RET agonist

## Abstract

**Background:**

Motor symptoms of Parkinson's disease (PD) are caused by degeneration and progressive loss of nigrostriatal dopamine neurons. Currently, no cure for this disease is available. Existing drugs alleviate PD symptoms but fail to halt neurodegeneration. Glial cell line–derived neurotrophic factor (GDNF) is able to protect and repair dopamine neurons *in vitro* and in animal models of PD, but the clinical use of GDNF is complicated by its pharmacokinetic properties. The present study aimed to evaluate the neuronal effects of a blood‐brain‐barrier penetrating small molecule GDNF receptor Rearranged in Transfection agonist, BT13, in the dopamine system.

**Methods:**

We characterized the ability of BT13 to activate RET in immortalized cells, to support the survival of cultured dopamine neurons, to protect cultured dopamine neurons against neurotoxin‐induced cell death, to activate intracellular signaling pathways both *in vitro* and *in vivo* *,* and to regulate dopamine release in the mouse striatum as well as BT13's distribution in the brain.

**Results:**

BT13 potently activates RET and downstream signaling cascades such as Extracellular Signal Regulated Kinase and AKT in immortalized cells. It supports the survival of cultured dopamine neurons from wild‐type but not from RET‐knockout mice. BT13 protects cultured dopamine neurons from 6‐Hydroxydopamine (6‐OHDA) and 1‐methyl‐4‐phenylpyridinium (MPP^+^)–induced cell death only if they express RET. In addition, BT13 is absorbed in the brain, activates intracellular signaling cascades in dopamine neurons both *in vitro* and *in vivo*, and also stimulates the release of dopamine in the mouse striatum.

**Conclusion:**

The GDNF receptor RET agonist BT13 demonstrates the potential for further development of novel disease‐modifying treatments against PD. © 2019 The Authors. *Movement Disorders* published by Wiley Periodicals LLC. on behalf of International Parkinson and Movement Disorder Society.

Parkinson's disease (PD) affects 1% to 2% of the population older than 60 years of age.^1^, ^2^ The characteristic motor symptoms of PD result from the progressive degeneration of dopamine neurons in the brain.^3^, ^4^ PD patients also suffer from multiple nonmotor symptoms.^4^, ^5^, ^6^ The current drugs for PD management are either supplementing or mimicking endogenous dopamine. Although these treatments provide symptomatic relief, they neither halt nor reverse the disease progression and have very little effect on nonmotor symptoms. Therefore, the major challenge remains to develop treatments that protect and restore dopamine neurons in PD patients and alleviate nonmotor symptoms.

Glial cell line‐derived neurotrophic factor (GDNF) and the related protein neurturin (NRTN), belonging to the GDNF family ligands (GFLs), promote the survival of cultured nigrostriatal dopamine neurons and protect and repair them in animal models of PD.^7^, ^8^, ^9^, ^10^, ^11^, ^12^, ^13^ GDNF and NRTN were also tested in patients with PD, but the results of these trials are controversial.^14^, ^15^, ^16^, ^17^, ^18^, ^19^, ^20^, ^21^, ^22^ Recently, it was shown that both intracranially infused GDNF and adeno‐associated virus vector encoded GDNF increased putamenal ^18^Fluoro‐dopamine uptake suggesting a neurotrophic effect of GDNF in the treated patients.^14^, ^20^, ^21^, ^23^ Furthermore, in the recent phase II trial, a post hoc analysis found 9 (43%) patients only in the GDNF‐treated group with a large clinically important motor improvement in the OFF state.^14^, ^20^, ^21^ It should also be noted that early‐stage PD patients, in contrast to late‐stage patients, might respond to adeno‐associated virus vector encoded NRTN treatment.^24^

The clinical utility of GFLs is complicated by their poor pharmacokinetic properties, necessity for intracranial delivery via stereotaxic surgery, varying biological activity, and high price. However, blood‐brain barrier penetrating small‐molecule compounds that target the GFL receptor complex, which consists of receptor tyrosine kinase REarranged during Transfection (RET) and GDNF family receptor α (GFRα),^25^ and mimic GFL biological effects in dopamine neurons may more easily translate to the clinic. Such compounds in principle can affect both terminal regions of dopamine neurons inducing sprouting and their cell bodies promoting survival, thus producing superior effects in comparison to GDNF and also alleviate the nonmotor symptoms of PD often caused by degeneration or dysfunction of GFL‐responsive brain and peripheral neurons.^26^, ^27^

Previously, we discovered a small molecule, BT13, that selectively activates GFL receptor‐dependent signaling in immortalized cells, supports sensory neurons, and alleviates neuropathy in rats.^28^ In the present study, we evaluated the biological effects of BT13 in the dopamine system. We demonstrate that BT13 activates RET receptor and supports the survival of cultured dopamine neurons RET‐dependently. It protects RET‐expressing cultured dopamine neurons from 6‐hydroxydopamine (6‐OHDA) and 1‐methyl‐4‐phenylpyridinium (MPP+)–induced cell death. In addition, BT13 activates intracellular signaling cascades in dopamine neurons both *in vitro* and *in vivo* and stimulates dopamine release in the mouse striatum. Our results indicate that BT13 can pave a way to the development of a novel treatment for PD.

## Materials and Methods

Experimental procedures are described in detail in the Supporting Information.

### Cell Lines

MG87, MG87RET cells,^29^ and reporter cell lines created on their basis were described previously.^30^

### Plasmids

Full‐length human(h) GFRα1 in pcDNA6,^30^ GFRα2 cDNA in pCR3.1 and enhanced green fluorescent protein (GFP) cDNA in pEGFP‐N1.

### Proteins

Human recombinant GDNF for *in vitro* experiments from Icosagen AS (Tartu, Estonia), and for *in vivo* studies from PeproTech (Rocky Hill, NJ, USA), fibroblast growth factor 2 (Basic Fibroblast Growth Factor) from BioVision Inc. (Milpitas, CA, USA).

### BT13

BT13 was synthesized by EvoBlocks Ltd. (Budapest, Hungary).

### Experimental Animals

All animal experiments were carried out according to the European Community guidelines for the use of experimental animals and approved by the National Animal Experiment Board of Finland (license numbers ESAVI/7551/04.10.07/2013 and ESAVI/198/04.10.07/2014) for experiments with living animals and the Laboratory Animal Centre of the University of Helsinki (license number KEK15‐022) for collection of E13.5 embryos of NMRI mice.

### Genotyping

Mouse embryonic day 13.5 (E13.5) embryos were genotyped for the presence or absence of RET as described previously.^31^

### Luciferase Assay

Luciferase assay was performed as described previously.^28^, ^30^

### Phosphorylation Assays

RET, ERK, and AKT phosphorylation in response to BT13 was analyzed in MG87RET cells transfected with hGFRα1, hGFRα2, and GFP‐expressing plasmids as described previously.^28^, ^32^

### Survival of Naïve and 6‐OHDA and MPP^+^ ‐Challenged Wild‐Type and RET‐Deficient Dopamine Neurons

The effect of BT13 on the survival of näive, 6‐OHDA, or MPP^+^ ‐challenged dopamine neurons was assessed using methods described previously.^33^, ^34^, ^35^, ^36^, ^37^, ^38^

### Analysis of phosphorylated ERK (pERK), AKT (pAKT), and Phospho‐Ribosomal Protein S6 (pS6) Levels in the Cultured Dopamine Neurons Treated With BT13 or GDNF

Midbrain neuron cultures were prepared as described previously.^33^, ^34^, ^36^ At 48 hours postplating, cultures were starved for 4 hours and treated with 1 μM of BT13 and 10 ng/ml of GDNF for 5 minutes for phosphorylated (p) ERK activation and 1 hour for phosphorylated (p) AKT. The cells were fixed and probed with tyrosine hydroxylase (TH), pERK, pAKT, and phospho‐ribosomal protein S6 (pS6, downstream target of AKT) antibodies.^28^, ^33^ The mean intensity of pERK, pAKT, and pS6‐specific staining in TH‐positive cells was measured using ImageJ software (Media Cybernetics Inc. [Rockville, MD, USA]) and normalized to the area of dopamine neuron where intensity was measured.

### Analysis of pERK and pS6 Levels in the Mouse Brains Treated With BT13 or GDNF

BT13 (103.5 μg [≈100 μg], N = 4; 207 μg [≈200 μg], N = 4; 517.5 μg [≈500 μg], N = 4; and 776.25 μg [≈750 μg], N = 4) or GDNF (5 or 10 μg [N = 4]) in saline with 0.5% DMSO (vehicle) were stereotactically injected into the left striatum using similar procedure as for *in vivo* microdialysis.^39^ The right striatum was injected with the vehicle. The striatal brain sections collected 1 hour after the injection were probed with pERK and pS6 antibodies.^28^ Optical density of pERK and pS6‐specific staining was measured in both hemispheres using ImageJ software (Media Cybernetics Inc) and normalized to the area of the analyzed selection. For presentation purposes, the values for the BT13‐treated and GDNF‐treated sides were normalized to the values for vehicle‐treated side of the same brain.

### *In Vivo* Microdialysis

*In vivo* microdialysis was performed exactly as described previously.^39^

### Pharmacokinetics of BT13 and Its Effects on the Levels of Dopamine Metabolites in the Midbrain

An analysis of BT13 concentrations in the brain and plasma at 0.5 to 2 hours postintravenous administration was conducted by Pharmidex Pharmaceutical Services Ltd (London, UK) using Ultra High Performance Liquid Chromatography/Liquid Chromatography‐Mass Spectrometry (UHPLC/LCMS) methods. The concentration of dopamine, 4‐dihydroxyphenylacetic acid (DOPAC), and homovanillic acid (HVA) was measured in midbrain samples by High Performance Liquid Chromatography.

### Statistical Analysis

The data were subjected to statistical analysis using the Student's *t* test or 1‐way analysis of variance (ANOVA) with a Dunnett's post hoc test in GraphPad Prism 6 (GraphPad Software Inc. [San Diego, CA, USA]).

## Results

### BT13 Stimulates RET Phosphorylation and Downstream Intracellular Signaling in Immortalized Cells

Dopamine neurons respond to GDNF^10^ and NRTN^40^ that signal via GFRα1/RET and GFRα2/RET, respectively.^25^ We evaluated the ability of BT13 to activate and signal via GFL receptors.

**Figure 1:**
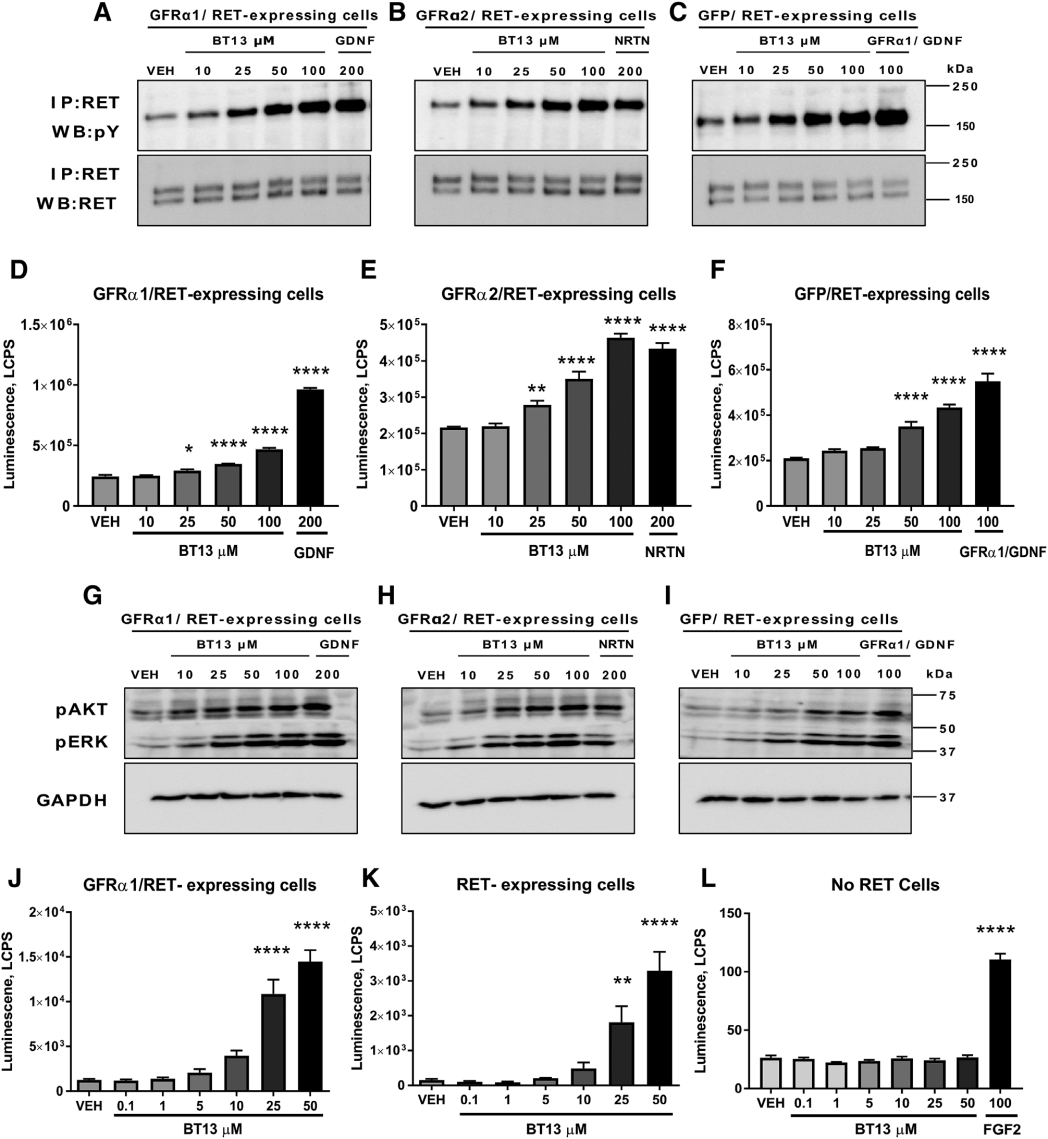
BT13 increases phosphorylation of RET (**A–C, D–F**) and its downstream targets AKT and ERK (**G–I, J, K**) in immortalized MG87RET fibroblasts, but shows no effect in reporter cell line lacking RET (**L**). Assessment of RET (A–C) and AKT/ERK phosphorylation by WB (G–I), phospho‐RET‐Enzyme‐Linked Immunosorbent Assay (ELISA) assay to quantify the level of RET phosphorylation (D–F) and activation of luciferase reporter controlled via ERK by RET (J,K) and fibroblast growth factor receptor (L). Effect of BT13 in MG87RET fibroblasts transfected with GFRα1 (A, D, G, J), GFRα2, (B, E, H) and GFP (C, F, I, K). Position of molecular weight markers (in kilodaltons, kDA) are shown on the right in A to C and G to I. Concentrations of GDNF, NRTN, and soluble GFRα1/GDNF complex used as positive controls for GFRα1, GFRα2, and GFP‐transfected MG87RET cells, respectively, and FGF2 in MG87 fibroblast cells are provided in ng/ml. One‐way analysis of variance with Dunnett's post hoc test. Mean ± standard error of mean. Number of experiments (N) = 3 to 4. FGF2, fibroblast growth factor 2; GAPDH, glyceraldehyde‐3‐phosphate dehydrogenase, a house‐keeping protein, loading control; GDNF, glial cell line–derived neurotrophic factor; GFP, green fluorescent protein; IP, immunoprecipitation; LCPS, luminescence counts per second; NRTN, neurturin; pERK, phosphorylated ERK; VEH, vehicle; WB, Western blotting. *P < 0.05, **P < 0.01, ***P < 0.0001.

In the cells expressing GFRα1/RET, 25 μM BT13 increased the level of phosphorylated RET by 1.2 fold (*P* = 0.0116), 50 μM by 1.4 fold (*P* < 0.0001), and 100 μM by 1.9 fold (*P* < 0.0001). In the cells expressing GFRα2/RET, 25 μM BT13 increased the level of pRET by 1.3 fold (*P* = 0.0088), 50 μM by 1.6 fold (*P* < 0.0001), and 100 μM by 2.1 fold (*P* < 0.0001). In the cells expressing GFP/RET, an increased level of pRET was observed in response to 50 μM (1.7‐fold increase, *P* < 0.0001) and 100 μM (2.1‐fold increase, *P* < 0.0001, 1‐way ANOVA with Dunnett's post hoc test for all comparisons) BT13 (Fig. 1A‐F). Cells responded in the expected manner to GDNF, NRTN, or soluble GFRα1/GDNF complex which were used as positive controls.

GDNF‐dependent and NRTN‐dependent autophosphorylation of RET causes activation of downstream signaling pathways, in particular ERK and AKT,^25^ important for the survival, regeneration, and functioning of neurons. BT13 activated both downstream targets in presence (GFRα1 transfected and GFRα2 transfected) and absence (GFP transfected) of GFRα coreceptors (Fig. 1G–I). Cells responded in the expected manner to GDNF and NRTN used as positive controls. To provide a quantitative estimate on the level of intracellular signaling cascade stimulation in response to BT13, we used luciferase reporter gene‐based assay reflecting the level of ERK activation.^30^ Consistent with Western blotting (Fig. 1G–I) and RET phosphorylation data (Fig. 1A–F), 25 and 50 μM BT13 increased luciferase activity in the reporter cell lines expressing GFRα1/RET by 8.6 fold and 11.5 fold, respectively (*P* < 0.0001) and in only RET‐expressing cells by 11.9 fold (*P* = 0.0040) and 21.7 fold, respectively (*P* < 0.0001, ANOVA with Dunnett's post hoc test in all cases; Fig. 1J,K). The difference in the amplitude of response to BT13 in GFRα1/RET and RET is likely caused by technical issues in assay setup (low basal activity of the reporter) as it was not observed in other assays. It is important to note that in the luciferase assay, GDNF usually increases reporter activity in GFRα1/RET–expressing cells by 50 to 100 fold.^28^, ^30^, ^33^ In the reporter cell line lacking RET, BT13 failed to influence luciferase activity while these cells responded as expected to a cognate inducer, FGF2 (4‐fold increase in luciferase activity, *P* < 0.0001).

### BT13 Supports the Survival of Cultured Dopamine Neurons

**Figure 2:**
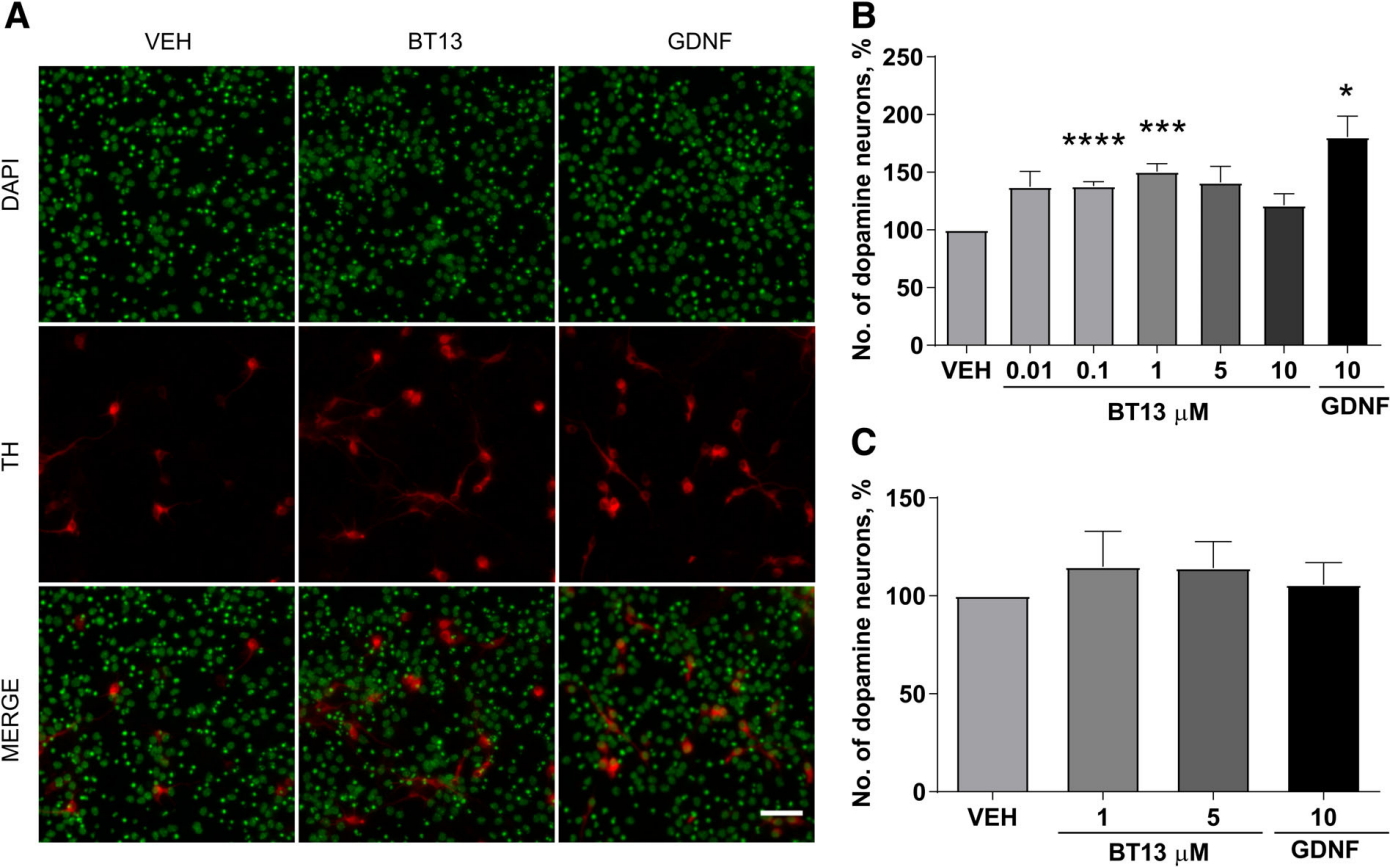
BT13 similarly to GDNF promotes the survival of cultured primary midbrain dopamine neurons from wild‐type (**A, B**), but not from RET‐knockout mice (**C**). (**A**) Representative images of mouse E13.5 wild‐type midbrain cultures treated with vehicle, BT13, and GDNF probed with anti‐TH antibody, pseudocolors. (**B**) The number of TH‐positive cells in the wild‐type midbrain cultures on the fifth Day in Vitro normalized to the total number of cells in the culture and presented as percentage of vehicle treated samples. (**C**) The number of TH‐positive cells in the RET‐knockout midbrain cultures on the fifth DIV normalized to the total number of cells in the culture and presented as percentage of vehicle treated samples, average from 3 experiments. Concentration of GDNF used as a positive control is provided in ng/ml. RM analysis of variance with Dunnett's post hoc test, mean ± standard error of the mean. Number of independent experiments (N) = 8. Scale bar = 50 μm. DAPI, 4′,6‐diamidino‐2‐phenylindole; GDNF, glial cell line–derived neurotrophic factor; VEH, vehicle. *P < 0.05, ***P < 0.001, ****P < 0.0001.

BT13 similarly to GDNF^10^ can promote the survival of cultured dopamine neurons. BT13 increased the number of TH‐positive cells in wild‐type embryonic midbrain culture by 1.4 fold (0.1 μM, *P* < 0.0001) and 1.5 fold (1 μM, *P* = 0.0002) and GDNF (10 ng/ml) by 1.8 fold (*P* = 0.0207, Repeated Measures (RM) ANOVA with Dunnett's post hoc test for all comparisons; Fig. 2A,B). It is important to note that the bell‐shaped dose‐response curves are common for neurotrophic factors in general and for GDNF in particular.^33^, ^41^ Both BT13 and GDNF failed to influence the survival of RET‐knockout embryonic midbrain dopamine neurons (Fig. 2C), therefore indicative of their selectivity toward RET.

### BT13 Protects Cultured Dopamine Neurons From 6‐OHDA and MPP^+^ Neurotoxicity Only if They Express RET

**Figure 3:**
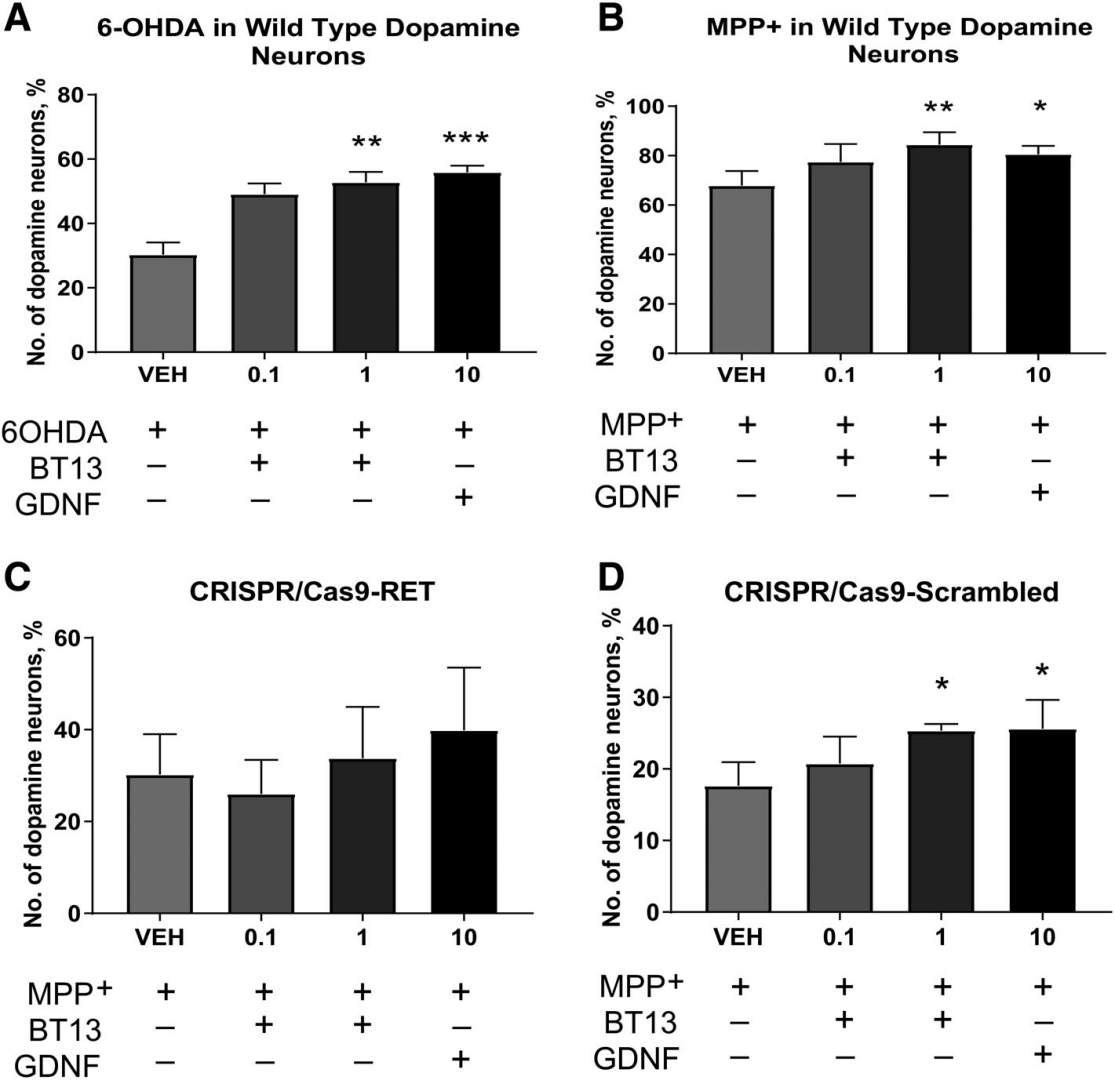
BT13 protects cultured dopamine neurons from 6‐OHDA (**A**) and MPP^+^ (**B**) neurotoxin‐induced cell death only when they express RET. The number of TH‐positive cells in the wild‐type midbrain cultures treated with 6‐OHDA (**A**) or MPP^+^ (**B**). The number of TH‐positive cells in midbrain neuron cultures where RET was deleted using CRISPR/Cas9 gene editing utilizing guide RNA targeting mouse RET (**C**) or scrambled guide (**D**). In (**A**) and (**B**), the toxins were added on the 6th DIV, and the number of TH‐positive cells was analyzed on the 8th DIV. In (**C**) and (**D**), lentiviral vectors were applied on the 1st DIV, MPP^+^ on the 8th DIV, and analysis was performed on the 10th DIV. In all cases, the number of TH‐positive cells was normalized to the total number of cells in the culture and presented as percentage of vehicle‐treated samples. All experiments were repeated 3 to 6 times with reproducible results. Concentration of BT13 is in μM and that of GDNF used as a positive control is provided in ng/ml. One‐way analysis of variance with Dunnett's post hoc test. Mean ± standard error of the mean. Number of wells (N) = 4 to 6 (**A**, **B**). Number of independent experiments (N) = 4 (**C**, **D**). GDNF, glial cell line–derived neurotrophic factor; MPP+, 1‐methyl‐4‐phenylpyridinium; VEH, vehicle. *P < 0.05, **P < 0.01, ***P < 0.001.

We assessed the neuroprotective ability of BT13 in cultured dopamine neurons treated with dopaminergic toxins. Treatment with 6‐OHDA and MPP^+^ decreased the number of survived TH‐positive cells in culture by 70% and 30%, respectively. BT13 similarly to GDNF protected embryonic midbrain dopamine neurons in culture from both 6‐OHDA‐induced and MPP^+^ ‐induced cell death (Fig. 3A,B). In the 6‐OHDA‐treated cultures, 0.1 and 1 μM BT13 increased the number of TH‐positive neurons by 1.6 fold (*P* = 0.0091) and 1.7 fold (*P* = 0.0026), respectively, and GDNF (10 ng/ml) by 1.8 fold (*P* = 0.0008, 1‐way ANOVA with Dunnett's post hoc test for all comparisons).

Similarly, in the MPP^+^ toxin model, BT13 (1 μM) increased the number of TH‐positive neurons by 1.3 fold (*P* = 0.0068) and GDNF (10 ng/ml) by 1.2 fold (*P* = 0.0389, 1‐way ANOVA with Dunnett's post hoc test for all comparisons). In an independent experiment carried out by Neuron Experts Company (Marseilles, France) in MPP^+^ ‐challenged rat E15 dopamine neurons, BT13 also increased the number of surviving TH‐positive neurons by 13% to 15% (data not shown).

The deletion of RET from cultured dopamine neurons using Clustered Regularly Interspaced Short Palindromic Repeats/CRISPR associated protein 9 (CRISPR/Cas9)‐mediated gene editing led to the loss of neuroprotection from the MPP^+^ lesion of both BT13 and GDNF (Fig. 3C). Importantly, in dopamine neurons treated with CRISPR/Cas9 lentiviral vector carrying scrambled guide both BT13 and GDNF remained neuroprotective (Fig. 3D) and increased the number of TH‐positive neurons by 1.7 fold (*P* = 0.0306 and *P* = 0.0254, respectively, 1‐way ANOVA with Dunnett's post hoc test).

### BT13 Stimulates Intracellular Signaling Important for the Survival and Regeneration of Cultured Dopamine Neurons

**Figure 4:**
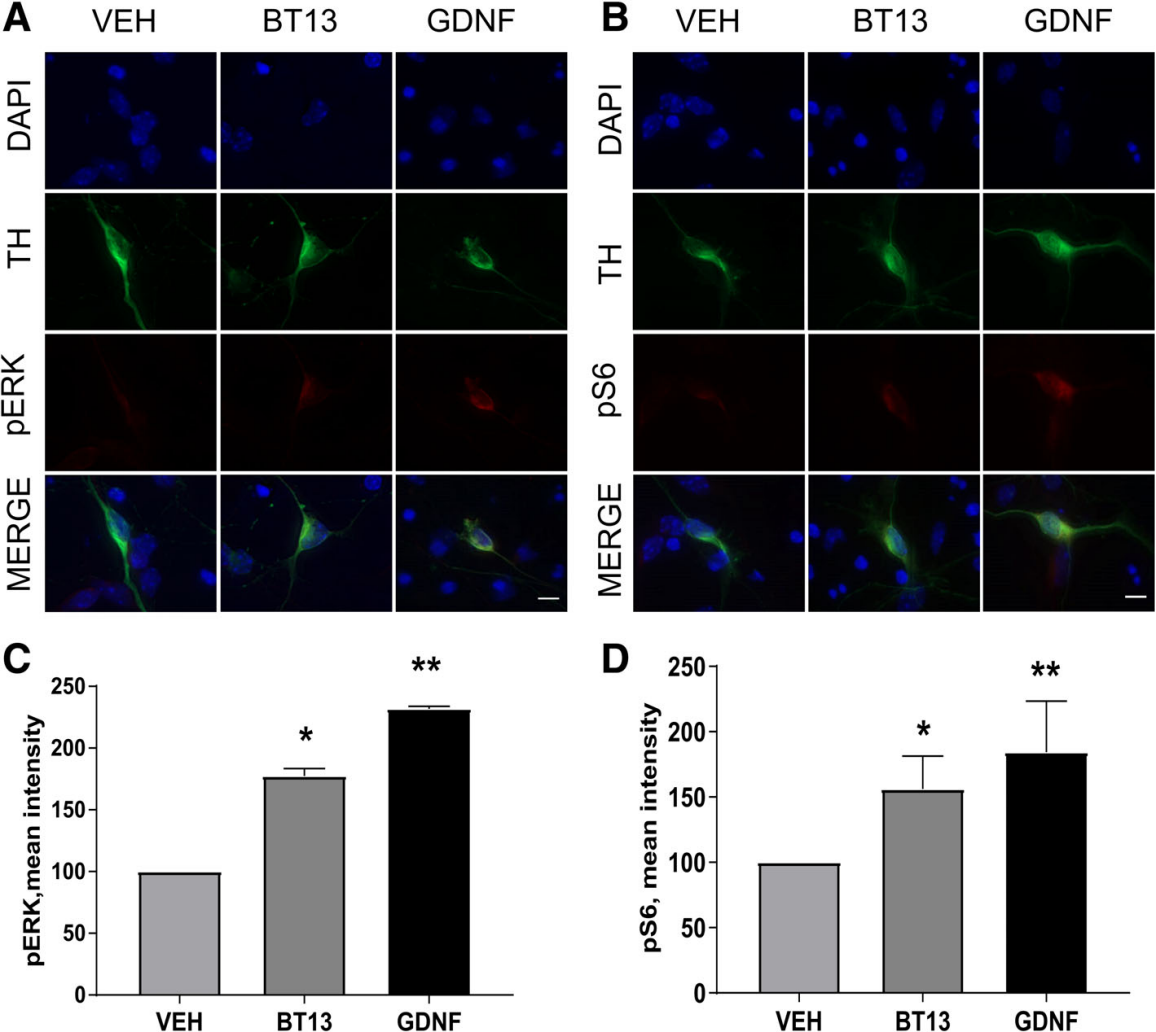
BT13 activates intracellular signaling cascades responsible for the survival and regeneration of neurons *in vitro*. Representative images of dopamine neurons probed with anti‐phospho‐ERK (pERK) (**A**) and with anti‐phospho‐S6 (pS6) antibody (**B**). (**C**) Mean intensity of pERK immunostaining in dopamine neurons in different treatment groups presented as percentage of vehicle‐treated dopamine neurons. (**D**) Mean intensity of pS6 immunostaining of dopamine neurons in different treatment groups presented as percentage of vehicle‐treated dopamine neurons. GDNF concentration, 10 ng/ml; BT13, 1 μM. One‐way analysis of variance with Dunnett's post hoc test. Mean ± standard error of the mean. Scale bar = 30 nm. Number of independent experiments (N) = 3. GDNF, glial cell line–derived neurotrophic factor; VEH, vehicle. *P < 0.05, **P < 0.01.

We further measured the mean intensity of pERK, pAKT and pS6 (downstream target of AKT, for which immunostaining produces more robust results compared to AKT itself^42^) in cultured dopamine neurons. The duration of stimulation for each target (5 minutes for pERK and 1 hour for pAKT and pS6) was chosen in pilot experiment in which we studied the time course of ERK and S6 activation in response to GDNF (data not shown). BT13 (1 μM) increased the mean intensity of pERK (Fig. 4A,C) by 1.7 fold (1 μM, *P* = 0.0371) and pS6 (Fig. 4B,D) by 1.5 fold (*P* = 0.0307). GDNF increased the mean intensity of pERK by 2.3 fold (*P* = 0.0086) and pS6 by 1.7 fold (*P* = 0.0052, 1‐way ANOVA with Dunnett's post hoc test for all comparisons). We also observed an increase in the level of pAKT in response to both BT13 and GDNF similar to their effect on pS6 phosphorylation in two experiments (Fig. S2).

### BT13 Stimulates Intracellular Signaling Important for Neuronal Survival and Regeneration in the Mouse Striatum

**Figure 5:**
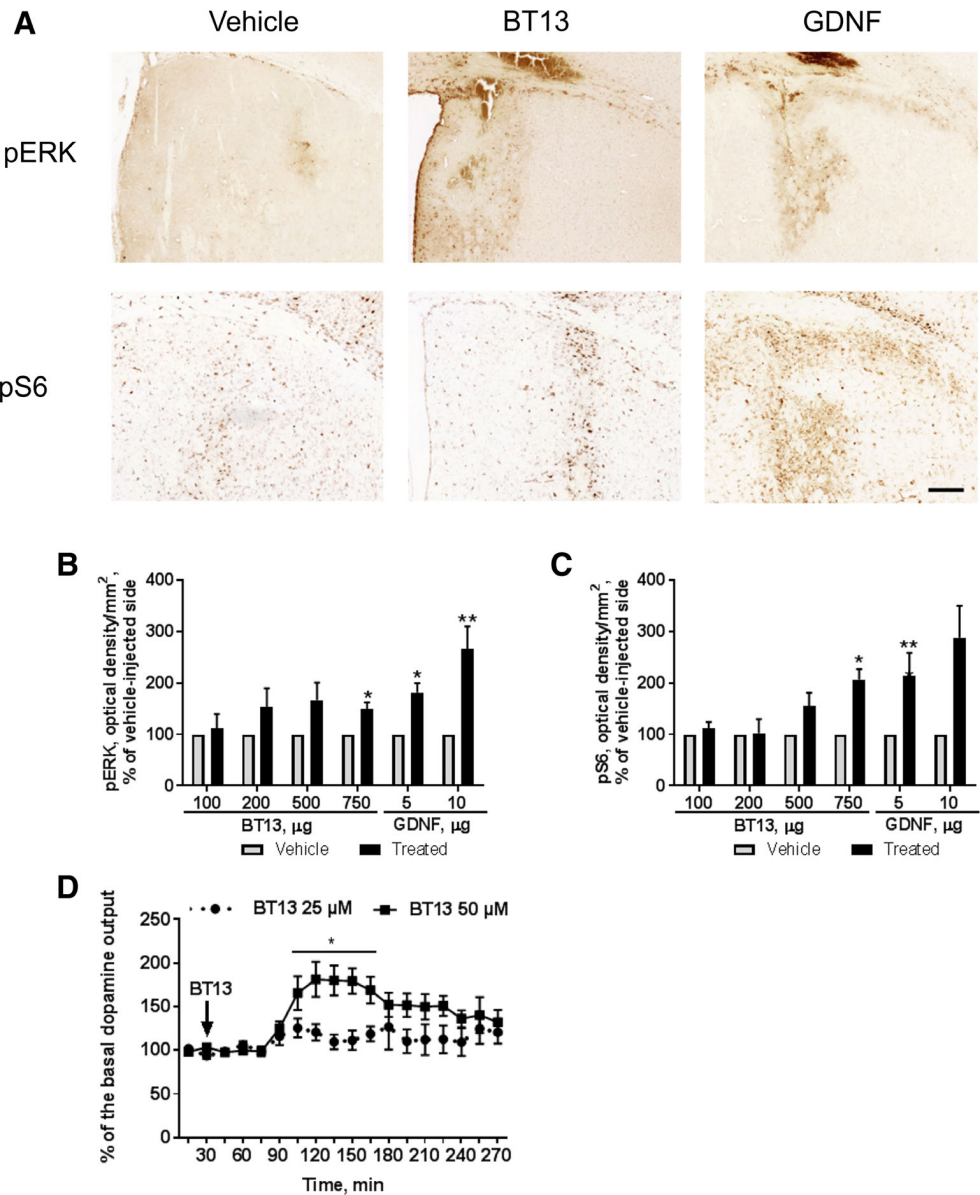
BT13 activates intracellular signaling cascades responsible for survival and regeneration of neurons *in vivo* and stimulates release of dopamine in mouse striatum. (**A**) Representative images of coronal sections from mouse brains probed with anti‐phospho‐S6 (pS6, lower pictures) and anti‐phospho‐ERK (pERK, upper pictures) antibodies. GDNF dose, 10 μg; BT13 dose, 750 μg. (B) Relative optical density of pERK immunostaining in the dorsal striatum in different treatment groups presented as percentage of vehicle‐treated side. (**C**) Relative optical density of pS6 immunostaining in the dorsal striatum in different treatment groups presented as percentage of vehicle‐treated side. Statistical significance for the differences between vehicle and BT13/GDNF‐injected sides was determined by paired 2‐tailed Student's *t* test. N = 3 to 4/group. Scale bar = 200 μm. (**D**) BT13 at 50 μM concentration increased extracellular dopamine level, whereas the lower concentration (25 μM) had no effect. One‐way analysis of variance with Dunnett's post hoc test. Mean ± standard error of the mean. Number of animals (N) = 5/group. GDNF, glial cell line–derived neurotrophic factor; VEH, vehicle. *P < 0.05, **P < 0.01. [Color figure can be viewed at wileyonlinelibrary.com]

We then measured the levels of pERK and pS6 in the mouse striatum after BT13 injection. Each mouse received an injection of a vehicle into the right striatum and BT13 or GDNF into the left striatum (Fig. 5A). The level of pERK and pS6 staining in vehicle‐treated striata remained unchanged between the treatment groups (*P* > 0.05, 1‐way ANOVA). BT13 at the dose of 750 μg increased phosphorylation of ERK and S6 compared to the vehicle‐treated striatum (*P* = 0.048 and *P* = 0.040, respectively, paired 2‐tailed Student's *t* test; Fig. 5B,C). GDNF at the dose of 5 μg elevated levels of both pERK (*P* = 0.0266) and pS6 (*P* = 0.0043) and at the dose of 10 μg increased phosphorylation of ERK (*P* = 0.0085) in the mouse striata.

### BT13 Stimulates Release of Dopamine in the Mouse Striatum

We tested whether BT13 similarly to GDNF^43^, ^44^, ^45^, ^46^ increases striatal dopamine release in mouse brain using brain microdialysis method. The infusion of 50 μM BT13 increased extracellular dopamine level in the striatum (Fig. 5D) at 105 to 165 minutes (30 minutes vs. 105 minutes *P* = 0.0160, 30 minutes vs. 120 minutes *P* = 0.0011, 30 minutes vs. 135 minutes *P* = 0.0013, 30 minutes vs. 150 minutes *P* = 0.0015, 30 minutes vs. 165 minutes *P* = 0.0091, 1‐way ANOVA with Dunnett's post hoc test) from the beginning of the experiment (or approximately 75–135 minutes after the beginning of BT13 infusion). It is important to note that the infusion of BT13 into the microdialysis system started at the 30‐minute timepoint, and it took 60 minutes for BT13 to pass through the tubing. Therefore, the effect of BT13 reached maximum 30 minutes after the entry of the first dose into the brain and remained at practically the same level for the next 30 minutes, followed by a gradual decrease. In the vehicle‐treated mice, the level of extracellular dopamine remained stable during the whole experiment (data not shown).

### BT13 Penetrates the Blood–Brain Barrier and Increases the Levels of Dopamine Metabolites in the Midbrain

We evaluated the concentration of BT13 in the frontal cortex, striatum, midbrain, cerebellum, and plasma 0.5 to 3 hours postintravenous administration. BT13 was detected in all 4 studied regions of the brain at concentrations exceeding the plasma's by approximately 1.2 to 3 fold (Table S1). An analysis of dopamine and dopamine metabolite content in the midbrain revealed a statistically significant increase in the concentration of HVA and a trend toward an increase in dopamine and DOPAC concentrations in rats receiving intravenous BT13 (Fig. S3).

## Discussion

We have characterized a small‐molecule RET agonist, BT13, in immortalized cells and in the dopamine system *in vitro* and *in vivo*. In immortalized cells, BT13 had similar efficacy to GFLs in RET, ERK, and AKT phosphorylation assays (Fig. 1A–I); however, in integral luciferase assay the effect of BT13 was much more modest compared with GDNF or soluble GDNF/GFRα1 complex (Fig. 1J,K^28^, ^30^). This can be partly explained by the quick metabolism of BT13.^28^ The efficacy of BT13 (100 nM and 1 μM) was comparable with that of GDNF in its ability to support the survival of cultured dopamine neurons (Fig. 2), to protect them from 6‐OHDA (Fig. 3A) and MPP^+^ ‐induced cell death (Fig. 3B), and to increase pERK and pS6 levels both *in vitro* (Fig. 4) and *in vivo* (Fig. 5). Nevertheless, the potency of BT13 was 2 to 3 orders of magnitude lower than that of GDNF, that is, GDNF supported the survival of cultured dopamine neurons at approximately the 0.3 nM concentration. Notably, the concentrations of the positive control proteins in all experiments were selected on the basis of previous results and biological activity in screening assay to produce clear effects in studied systems.^28^, ^30^, ^33^, ^47^, ^48^

Importantly, both BT13 and GDNF promoted the survival of the primary dopamine neurons in lower concentrations than in immortalized cells. Artificial cell systems differ from primary neurons in many ways. In particular, the stoichiometry of relevant receptors, intracellular mediators, and other factors may differ and thereby regulate the sensitivity to agonists. The limited aqueous solubility of BT13 prevented us from testing it in higher concentration (above 100 μM) in murine fibroblasts. Perhaps in immortalized cells the full potential of BT13 to promote phosphorylation of RET and intracellular signaling cascades was not achieved because of its insufficient solubility in the cell culture medium.

Earlier, we showed that BT13 activates RET selectively^28^; it is unable to stimulate other receptor tyrosine kinases, for example, Tropomyosin receptor kinase B (TrkB), a receptor for brain‐derived neurotrophic factor, which is a known survival factor for dopamine neurons; TrkB‐dependent activation of AKT and MAPK signaling cascades or activation of AKT and ERK in the MG87 parental cells.^49^ Consistent with these data, in the present study BT13 was unable to increase luciferase activity in the reporter cell line lacking RET, whereas FGF2 activated the reporter in these cells indicative of their functional integrity (Fig. 1L). We also showed that the survival promoting effect of BT13 in cultured dopamine neurons is RET‐dependent (Fig. 2B). BT13 similarly to GDNF is unable to promote the survival of naïve and toxin‐challenged dopamine neurons lacking RET (Fig. 2C and Fig. 3C). In addition, BT13 does not promote the survival of GABA‐positive neurons (Fig. S1), suggesting that BT13 is selective to dopamine neurons in midbrain cultures. However, in contrast to GDNF, BT13 is a direct RET agonist and it does not require a GFRα coreceptor to elicit biological effects (Fig. 1). Molecular dynamics simulation studies demonstrated that the compound can bind on the interface of RET and GFRα1.^50^ Therefore, BT13 can influence neurons expressing any combination of GFRα coreceptors and RET, thus increasing the proportion of responsive neurons.

The degeneration of dopamine neurons caused by 6‐OHDA and MPP^+^ is often used to model PD and evaluate neuroprotective properties of studied compounds *in vitro* and *in vivo*. GDNF protects and restores dopamine neurons in culture and in experimental animals upon treatment with either of these neurotoxins.^7^, ^8^, ^41^, ^51^, ^52^ In the present study, 1 μM BT13 similarly to GDNF showed significant neuroprotection of RET‐expressing dopamine neurons against both 6‐OHDA and MPP^+^ ‐induced degeneration (Fig. 3A,B). We also observed significant neuroprotective effect of BT13 at a lower concentration (0.1 μM) in 6‐OHDA‐treated dopamine neurons. In MPP^+^ ‐treated dopamine neurons the number of survived cells had a tendency to increase in response to 0.1 μM BT13, but this effect was not statistically significant (*P* = 0.19). It should be noted that the degeneration of dopamine neurons induced by MPP+ in our experiments was milder compared with the effect of 6‐OHDA (Fig. 3). Therefore, a small effect size could have prevented us from seeing significant effect of lower concentration of BT13 in MPP^+^ *in vitro* model of PD. Alternatively, this discrepancy can be explained by the fact that different toxin model might not respond in a similar way to different concentrations of BT13.

The neuroprotective and neurorestorative effects of GDNF in dopamine neurons are mediated by activation of intracellular pathways such as PI3K/AKT and MAPK, which play a role in cell survival and differentiation.^25^, ^53^, ^54^, ^55^, ^56^, ^57^ In the present study, we showed that 1 μM BT13 similarly to GDNF activated ERK and AKT pathways in cultured dopamine neurons (Fig. 4 and Fig. S2). It is important to note that 1 μM BT13 also promoted the survival of näive dopamine neurons (Fig. 2) and protected them from toxin‐induced death (Fig. 3). In addition, BT13 also significantly activated ERK and AKT (assessed by pS6 level) pathways when injected into the striatum of naïve animals (Fig. 5A–C). Taken together, these *in vitro* and *in vivo* results suggest that BT13 can play a potential role in the survival and regeneration of dopamine neurons.

Microdialysis data show that infusion of BT13 into the striatum increases extracellular dopamine level (Fig. 5D), further indicating its ability to activate relevant signaling pathways in the brain. Notably, GDNF overexpression from the native locus enhances dopamine release in the striatum of transgenic mice.^39^ Importantly, the ability of BT13 to elevate extracellular dopamine level may not correlate with its neurotrophic activity. The effect on dopamine level is possibly mediated through the inhibition of A‐type potassium channels that GDNF is known to regulate,^58^ but it may also be the consequence of dopamine transporter (DAT) regulation that was recently characterized for GDNF.^59^, ^60^ The fact that the extracellular dopamine level started to decrease after some time even though the infusion continued suggests activation of homeostatic mechanisms. These mechanisms might include alterations in the availability or activity of GDNF receptors and/or DAT.^61^

We showed that systemically administered BT13 gets into the brain that is in line with our previous findings^28^ and reaches the nigrostriatal dopamine system (Table S1). Moreover, it may influence dopamine content and metabolism in the midbrain (Fig. S3). The limited aqueous solubility of BT13 and its quick metabolism^28^ likely restricted its free concentration and duration of action in the brain after bolus injection. Therefore, we observed only a trend in some neurochemical parameters measured in our pilot experiment with relatively small number of animals per group. We noticed a statistically significant increase in HVA level accompanied by a slight increase in the HVA/DA ratio, which represents a sum of dopamine turnover and release,^62^ but no changes in the DOPAC/DA ratio, which is an indicator of dopamine turnover^63^ 30 minutes posttreatment with BT13. Taken together, these data suggest that BT13 affects dopamine release rather than dopamine synthesis, which is consistent with microdialysis experiment data (Fig. 5D). Based on the results of the pharmacokinetic and neurochemical studies, we can conclude that optimized BT13 derivatives with increased half‐life and better brain exposure should increase dopamine release in nigrostriatal system also after systemic administration.

In conclusion, our data offer a proof of principle for small‐molecule synthetic RET agonists in *in vitro* neurotoxin model of PD. Our first‐generation compound BT13 has promising effects on dopaminergic system both *in vitro* and *in vivo*. We are currently improving the potency and pharmacological properties of BT13 using medicinal chemistry methods to develop a lead compound to test in animal models of PD.

## Author Roles

Research Project: A. Conception, B. Organization, C. Execution, D. Overall Supervision; (2) Statistical Analysis: A. Design, B. Execution, C. Review and Critique; (3) Manuscript Preparation: A. Writing of the First Draft, B. Review and Critique.

A.K.M.: 1C, 2A, 2B, 3A, 3B

J.K.: 1C, 2A, 2B, 3A, 3B

J‐M.R.: 1C, 3B

T.V.: 1C, 3B

I.K.: 1C, 2B, 3B

N.P.: 1C, 2B, 3B

M.M.B.: 1A

A.D.: 1C, 3B

E.R.: 1C, 2B, 3B

T.P.P: 1B, 2C, 3B

R.K.T.: 1B, 2C, 3B

M.K.: 1A, 1C, 3B

Y.S.: 1A, 1B, 1D, 2A, 2C, 3A, 3B

M.S.: 1A, 1B, 1D, 2A, 2C, 3B M.H.V.: 1B, 2C, 3B

## Financial Disclosures of all authors (for the preceding 12 months):

none.

## Supporting information


**Appendix S1:** Supporting Information
