# Supplementary material for: Glial Cell Line–Derived Neurotrophic Factor Receptor Rearranged During Transfection Agonist Supports Dopamine Neurons In Vitro and Enhances Dopamine Release In Vivo
Source: Mov Disord. 2019 Dec 16;35(2):245–55. doi: 10.1002/mds.27943 (PMC7496767; doi:10.1002/mds.27943)
Supplement: Supplementary file 1 — Appendix S1: Supporting Information [file MDS-35-245-s001.pdf]

Title: GDNF receptor RET agonist supports dopamine neurons *in vitro* and enhances dopamine release *in vivo*

Arun Kumar Mahato[1], MSc., Jaakko Kopra[2], PhD, Juho-Matti Renko[2], MSc., Tanel Visnapuu[2], PhD, Ilari Korhonen[2], MSc., Nita Pulkkinen[2], MSc., Maxim M. Bespalov[1¤], PhD, Andrii Domanskyi[1], PhD, Eric Ronken[3], PhD, T. Petteri Piepponen[2], PhD, Merja Voutilainen[1], PhD, Raimo K. Tuominen[2], PhD, Mati Karelson[4], PhD, Yulia A. Sidorova[1#§], PhD, and Mart Saarma[1*§], PhD

Supplemental Material

Detailed description of materials and methods

Cell Lines: MG87 RET murine fibroblasts stably transfected with RET oncogene (1). MG87RET fibroblasts stably transfected with GFRα1-expressing plasmid or empty vector and a luciferase reporter gene system (PathDetect detect Elk-1 trans-Reporting system, Stratagene/Agilent Technologies, USA) to detect activation of mitogen-activated protein kinase (MAPK) signaling pathway (2).

Plasmids: Full-length human GFRα1 cDNA subcloned in pCDNA6 (Invitrogen, USA) (2), GFRα2 cDNA in pCR3.1 and enhanced green fluorescent protein (GFP) cDNA in pEGFP-N1.

Proteins: Human recombinant GDNF (hGDNF) for *in vitro* experiments was produced in mammalian CHO cells and obtained from Icosagen (Estonia), hGDNF for *in vivo* studies was produced in *E.coli* and was purchased from PeproTech (USA). FGF-2 was purchased from BioVision Inc (4037-50).

BT13: The synthetic route for the compound BT13 is given below

Step 1

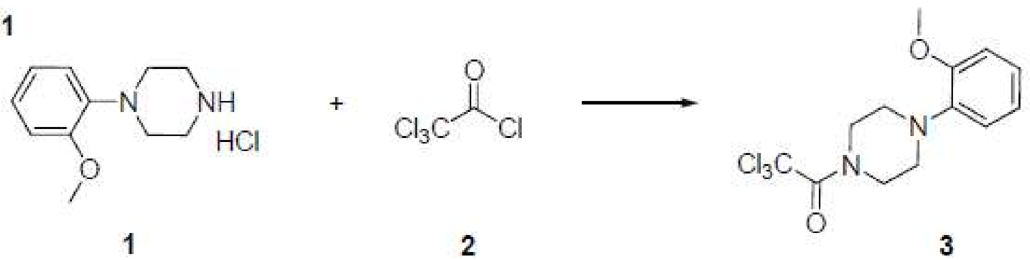

1 **2** **3**

[a] - After these studies were completed, Solvay was acquired by Abbott labs

Step 2

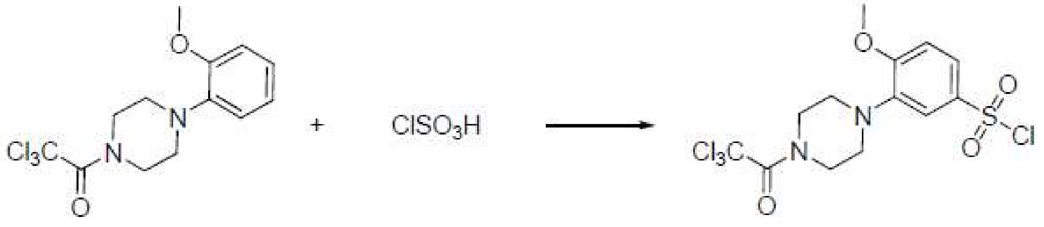

3 4 5

Step 3A

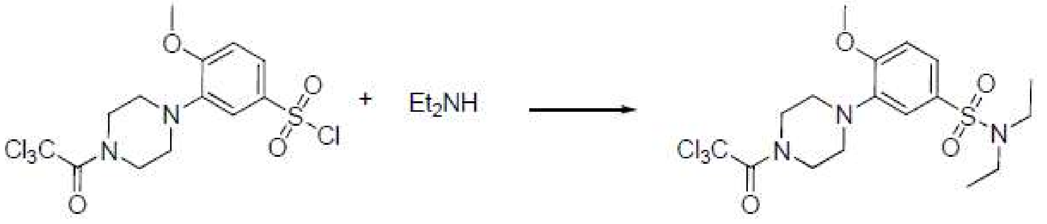

5 6 7

Step 4A

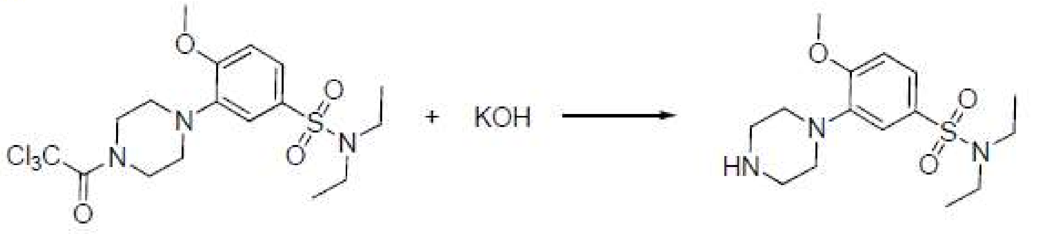

7 8

Step 5A

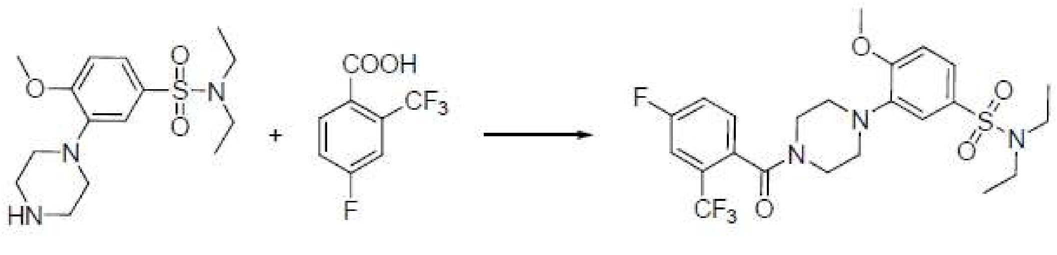

9 10 11

The individual steps of the synthesis were carried out as follows.

Synthesis of 1-Trichloroacetyl-4-(2-methoxyphenyl)piperazin (**3**)

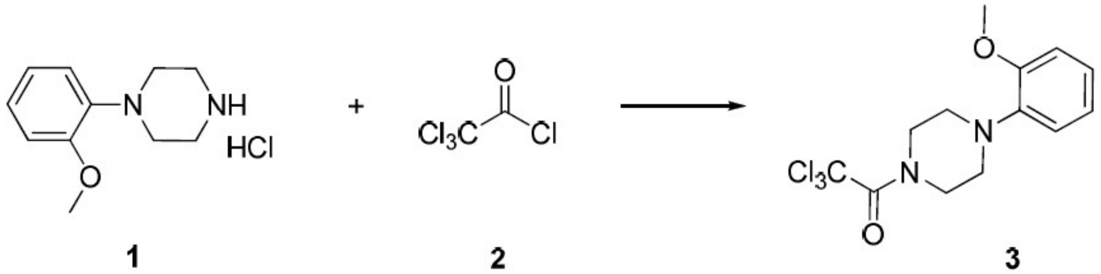

1 **2** **3**

In a 2-L round-bottom flask equipped with thermometer, mechanical stirrer, dropping funnel and drying tube 1-(2-methoxyphenyl)piperazin hydrochloride (**1**) (91.00 g, 398 mmol) was suspended in dry dichloromethane (1000 mL). With well stirring and keeping the temperature below 5 °C trichloroacetyl chloride (**2**) (46.60 ml, 418 mmol, 1.05 equiv.) was added dropwise followed by the addition of N,N-diisopropylethylamine (146 ml, 855 mmol, 2.15 equiv.). The reaction mixture was let to warm up to room temperature and the stirring was continued for an additional hour, by that time the reaction was completed.

Water (ca. 800 ml) was added to the reaction mixture and after a few minutes the phases were separated. The aqueous part was extracted once with dichloromethane, the combined organic phases were washed with water and brine, dried over $MgSO_4$ and evaporated. The crystalline residue was suspended in diisopropyl ether, filtered off and washed with the same solvent. The title product was dried in a vacuum desiccator over P_2O_5/KOH. Yield: 129.5 g of **3** (96%) as pale brown crystals.[5] **LU-240 (3):** ^{1}H NMR (200 MHz, *DMSO-*d_6) δ ppm 6.86 – 7.02 (m, 11H), 3.70 – 4.10 (m, 7H); APCI MS *m/z* 337 $[M + H]^+$; HPLC-MS >99.0% (AUC).

Synthesis of 4-methoxy-3-(4-trichloroacetylpiperazin-1-yl)benzenesulfonyl chloride (**5**)

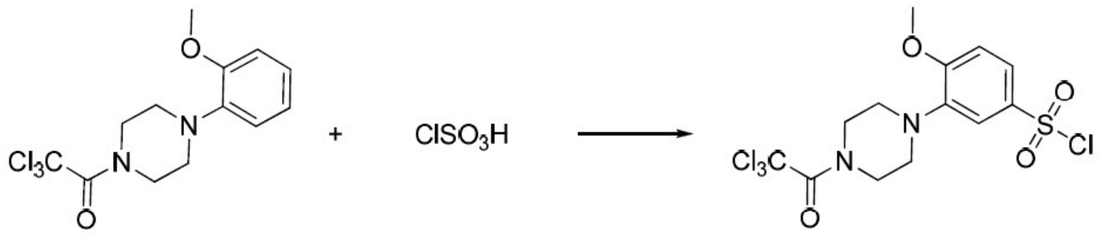

3 **4** **5**

To a 2-L round-bottom flask equipped with thermometer, mechanical stirrer, dropping funnel and drying tube chlorosulfonic acid (**4**) (1.14 kg, 9.78 mol, 0.65 L) was charged. With well stirring and cooling to keep the temperature below 5 °C the solution of the piperazine derivative (**3**) (129.0

g; 384 mmol) in dry dichloromethane (500 ml) was added dropwise. The reaction mixture was let to warm up to room temperature and the stirring was continued for an additional hour.

To a five liter beaker, equipped with thermometer, mechanical stirrer and immersed into a saltice cooling mixture ca. 1 kilogram of ice was placed, and then the reaction mixture was slowly poured into it such a rate, that the temperature kept below 5 °C. The phases were separated and the aqueous part was extracted once with dichloromethane. The combined organic phase was washed with water and brine, dried over $MgSO_4$ and evaporated to dryness. The crystalline residue was suspended in diisopropyl ether, filtered off and washed with the same solvent. Yield: 117.2 g of **5** (70%) as light brown crystals. **LU-238 (5):** ^{1}H NMR (200 MHz, *DMSO-d_6*) δ ppm 7.44 (s, 1H), 7.41 (d, J = 8.4 Hz, 1H), 7.03 (d, J = 8.4 Hz, 1H), 3.85 (s, 3H), 3.90 – 4.15 (m, 8H); APCI MS *m/z* 436 $[M + H]^+$; HPLC-MS 95.0% (AUC).

Synthesis of N,N-diethyl-4-methoxy-3-(4-trichloroacetylpiperazin-1-yl)benzenesulfonamide (**7**)

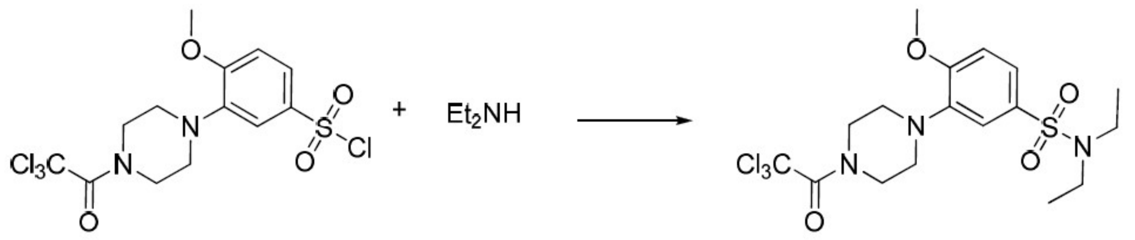

5 **6** **7**

In a 1-L round-bottom flask equipped with thermometer, magnetic stirrer, dropping funnel and drying tube 21.30 ml of diethylamine (**6**) (15.10 g, 206 mmol, 3.00 equiv) was diluted with dry dichloromethane (300 mL). With well stirring and keeping the temperature below 5 °C the solution of sulfochloride derivative (**5**) (30.00 g; 69 mmol.) in dry dichloromethane (200 ml) was added dropwise. After the addition had taken place the reaction mixture was let to warm up to room temperature and the stirring was continued for additional 30 min, by that time the reaction has been completed.

The reaction mixture was diluted with water and the phases were separated. The organic phase was washed twice with water and once with brine, dried over $MgSO_4$ and evaporated to dryness. The crystalline residue was suspended in diisopropyl ether, filtered off and washed with the same solvent. Yield: 31.30 g of **7** (96%) as light brown powder. **LU-247 (7):** ^{1}H NMR (200 MHz, *DMSOd$_6$*) δ ppm 7.44 (d, J = 8.4 Hz, 1H), 7.17 (s, 1H), 7.14 (d, J = 8.4 Hz, 1H), 3.70 – 4.10 (m, 11H), 3.11 (q, J = 7.2 Hz, 4H), 1.02 (t, J = 7.2 Hz, 6H); APCI MS *m/z* 472 $[M + H]^+$; HPLC-MS 96.0% (AUC).

Synthesis of N,N-diethyl-4-methoxy-3-(piperazin-1-yl)benzenesulfonamide (**8**)

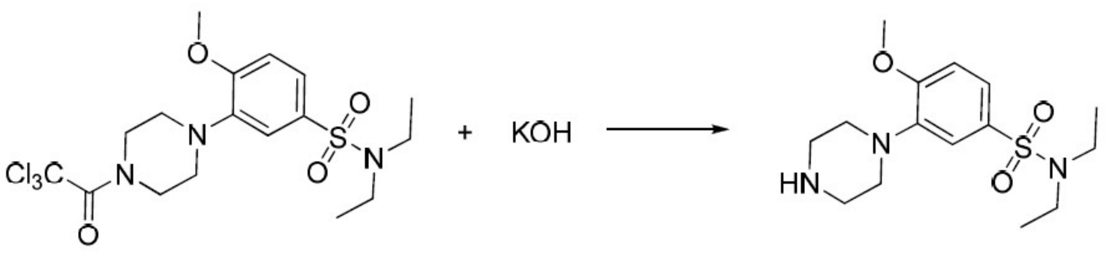

7 **8**

To a suspension of sulfonamide (**7**) (31.30 g, 66.2 mmol) in tetrahydrofuran (300 ml) was added the aqueous solution of potassium hydroxide (9.30 g, 166 mmol, 2.50 equiv., in 40 mL water) in one portion and the mixture was stirred at 40-50 °C for four hours. The same amount of potassium hydroxide (9.30 g, 166 mmol, 2.50 equiv) was dissolved in water (10 mL) and it was also added to the reaction mixture and the stirring was continued at 50 °C for another four hours then at room temperature overnight.

After completion of the reaction the solvent was evaporated, the aqueous residue was diluted with water and extracted with dichloromethane three times. The targeted piperazine derivative (**8**) was extracted from the organic phase three times with 3% HCl solution. The combined aqueous solution was cooled off, basified with 20% aqueous NaOH and extracted three times with dichloromethane. The combined organic solutions were washed with brine, dried over $MgSO_4$ and evaporated. The resulted yellow oil became crystalline on standing in refrigerator. It was suspended in a small amount of diisopropyl ether, filtered off and washed with the same solvent. Yield: 15.15 g of **8** (70%) as yellow crystalline. **LU-242 (8):** ^{1}H NMR (200 MHz, *DMSO-*d_6) δ ppm 7.34 – 7.48 (m, 1H), 7.05 – 7.20 (m, 2H), 3.86 (s, 3H), 2.90 – 3.20 (m, 12H), 2.61 (m, 1H), 1.02 (t, J = 7.2 Hz, 6H); APCI MS *m/z* 327 $[M + H]^+$; HPLC-MS >99.0% (AUC).

Synthesis of N,N-diethyl-3-(4-(4-fluoro-2-(trifluoromethyl)benzoyl)piperazin-1-yl)-4-methoxybenzenesulfonamide (**11**)

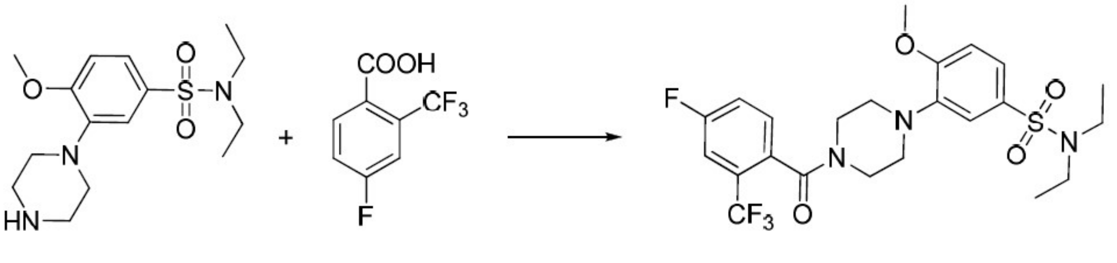

9 **10** **11**

To a suspension of 4-fluoro-2-(trifluoromethyl)benzoic acid (**10**) (9.53 g, 46 mmol) in tetrahydrofuran (400 ml) was added 1,1'carbonyldiimidazole (8.17 g, 50 mmol, 1.10 equiv.) and the reaction mixture was stirred at room temperature for 2 hours. The solution of the piperazine derivative (**9**) (15.00 g, 46 mmol) in a minimal amount of tetrahydrofuran was added in one portion and the reaction mixture was stirred at 50 °C for 20 hours.

After the reaction had completed the solvent was evaporated. The residue was dissolved in dichloromethane and was washed with 3% aqueous HCl solution, water, saturated aqueous $NaHCO_3$ solution and brine, dried over $MgSO_4$, filtered and finally evaporated to dryness. This crude product was recrystallized from hot, 50% aqueous ethanol (500 ml) using norit. Yield: 12.91 g of **11** (54%) as white powder. **LU-246 (11):** 7.78 (d, *J* = 8.4 Hz, 1H), 7.61 – 7.69 (m, 2H), 7.08 – 7.18 (m, 2H), 3.87 (s, 3H), 3.70 – 3.82 (m, 4H), 2.75 – 3.35 (m, 8H), 1.01 (t, *J* = 7.2 Hz, 3H); APCI MS *m/z* 517 $[M + H]^+$; HPLC-MS >99.0% (AUC).

Thin-layer chromatography (TLC) was performed using silica gel 60 F254 plates (Merck) and visualized by UV light (254 nm). Column chromatography was carried out on Biotage Horizon flash purification system using silica gel 20-40 unless otherwise specified. Proton nuclear magnetic resonance spectra were obtained on a Varian Unity 200 MHz instrument. For the calibration of spectra, solvent peak and tetramethylsilane signals were used. Spectra were performed at room temperature; the results are given in ppm (δ) with coupling constants and *J* values reported in hertz. The HPLC-MS analysis was performed on a Waters HPLC/MS (with 4-channel MUX interface) with a LiChroCART 30-4 Purospher STAR RP-18, endcapped, 3μm (Merck) column using a solvent gradient program.

BT13 NMR data:

NMR Chemical shifts of BT13 (850 M, 30 °C, *d6*-DMSO)

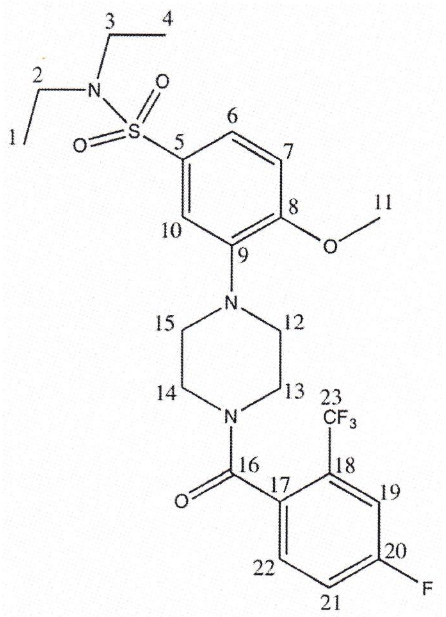

#	δ_{13C} [ppm]	J_{CF} Couplings [Hz]	δ_{1H} [ppm]
1	14.0		1.01
2	41.64		3.11
3	41.64		3.11
4	14.0		1.01
5	131.43		
6	122.29		7.41
7	111.7		7.12
8	155.0		
9	140.7		
10	116.2		7.14
11	55.85		3.86
12 & 15	49.45; 49.50		2.84 (ax), 2.97 (eq); 3.03 (ax), 3.08 (eq)
13 & 14	41.3; 46.7		3.19 (ax), 3.28 (eq); 3.73 (ax), 3.81 (eq)
16	165.3		
17	131.2	$^4J_{CF}$ 2.3	
18	127.5	$^2J_{CF}$ 32.6, $^3J_{CF}$ 7.8	
19	114.2	$^2J_{CF}$ 25.6, $^3J_{CF}$ 4.5	7.76
20	161.5	$^1J_{CF}$ 248	
21	120.0	$^2J_{CF}$ 21.2	7.63
22	130.35	$^3J_{CF}$ 8.2	7.63
23	122.79	$^1J_{CF}$ 273, $^4J_{CF}$ 2.4	

Tuomas Niemi-Aro, Harri Heikkinen
15.11.2017

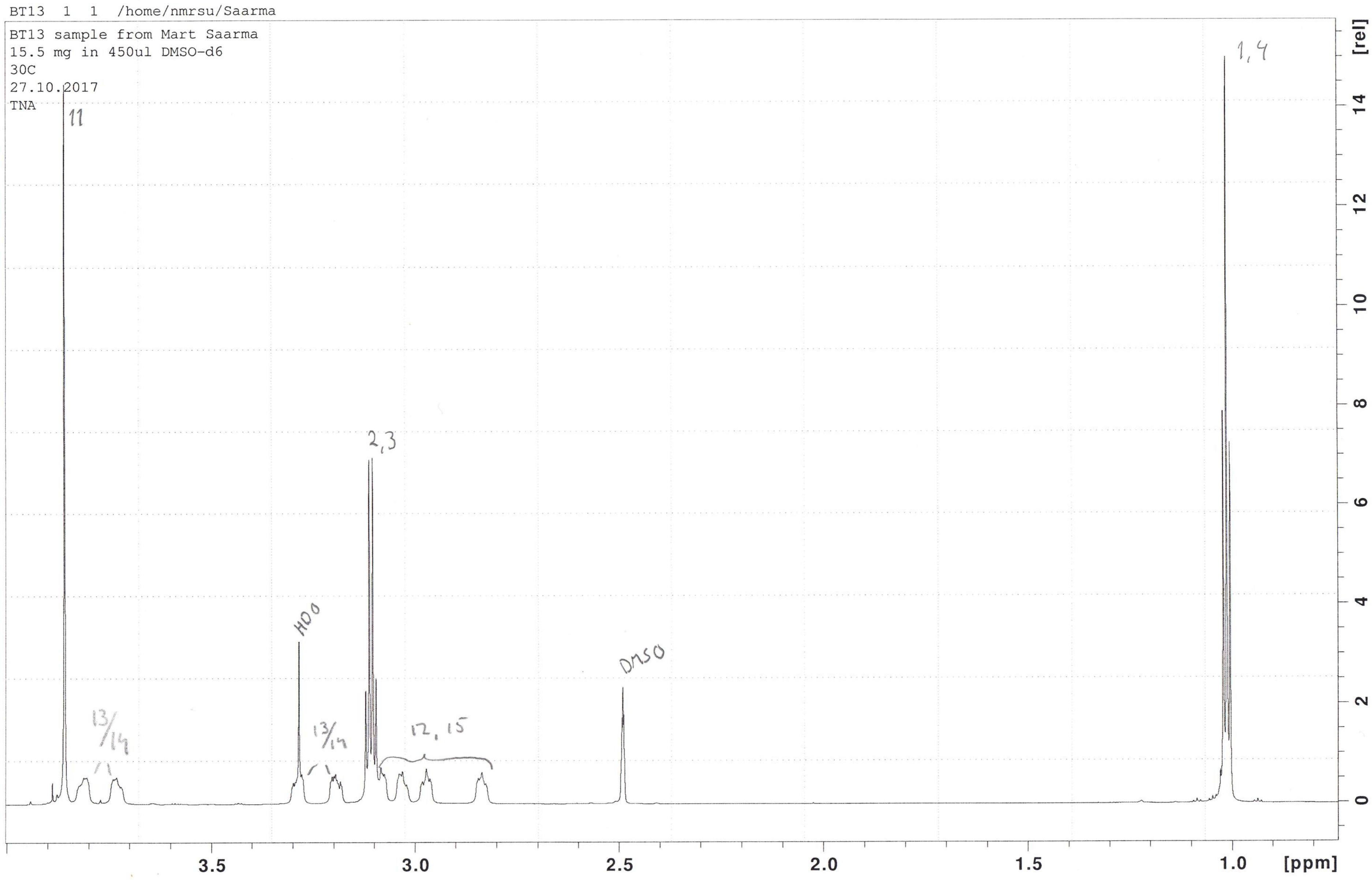
BT13 1 1 /home/nmrsu/Saarma
BT13 sample from Mart Saarma
15.5 mg in 450ul DMSO-d6
30C
27.10.2017
TNA
11
13/14
HDO
13/14
2,3
12, 15
DMSO
1,4
3.5
3.0
2.5
2.0
1.5
1.0
[ppm]
0
2
4
6
8
10
12
14
[rel]

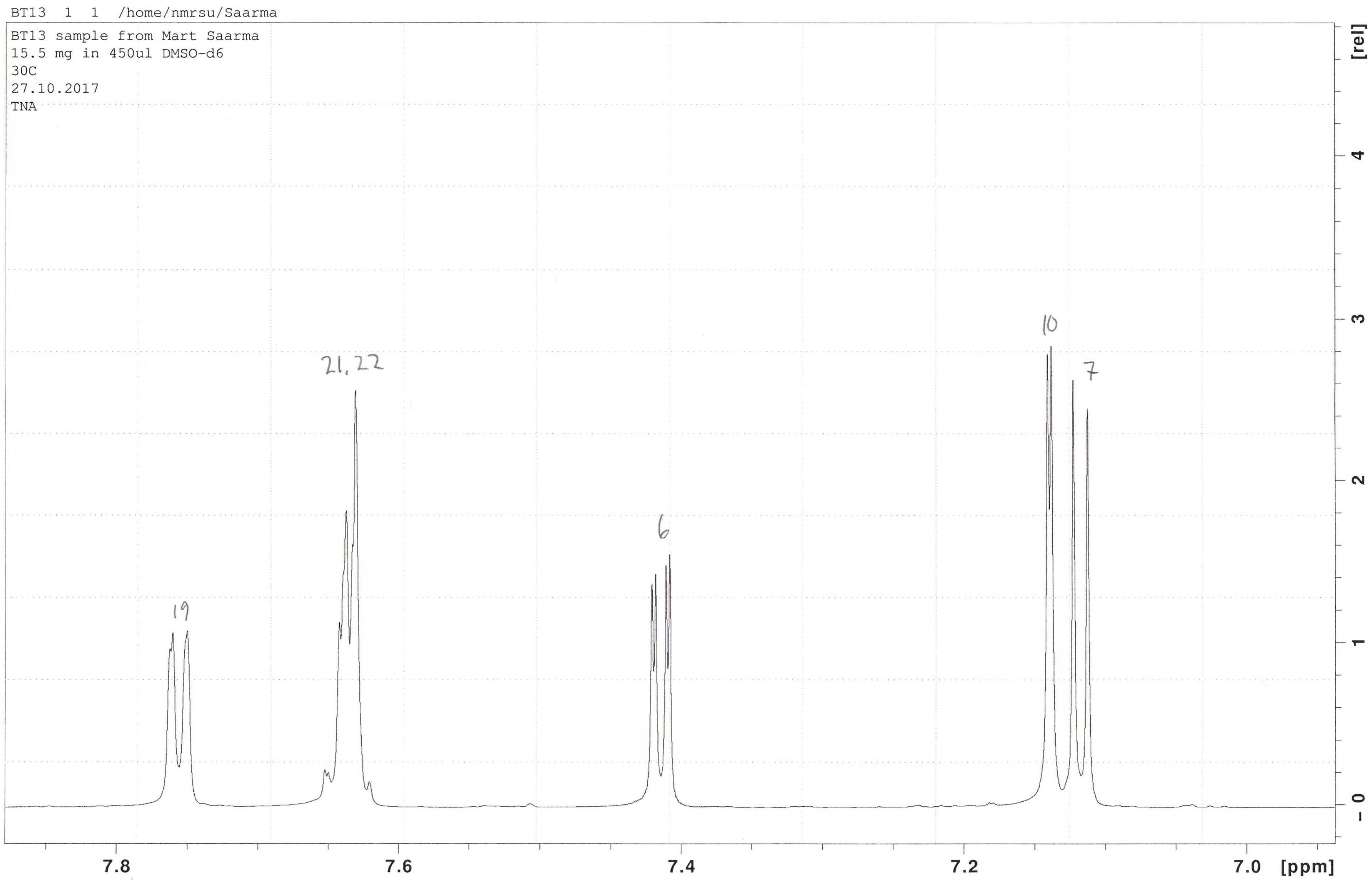
BT13 1 1 /home/nmrsu/Saarma
BT13 sample from Mart Saarma
15.5 mg in 450ul DMSO-d6
30C
27.10.2017
TNA
19
21,22
6
10
7
7.8
7.6
7.4
7.2
7.0
[ppm]
0
1
2
3
4
[rel]

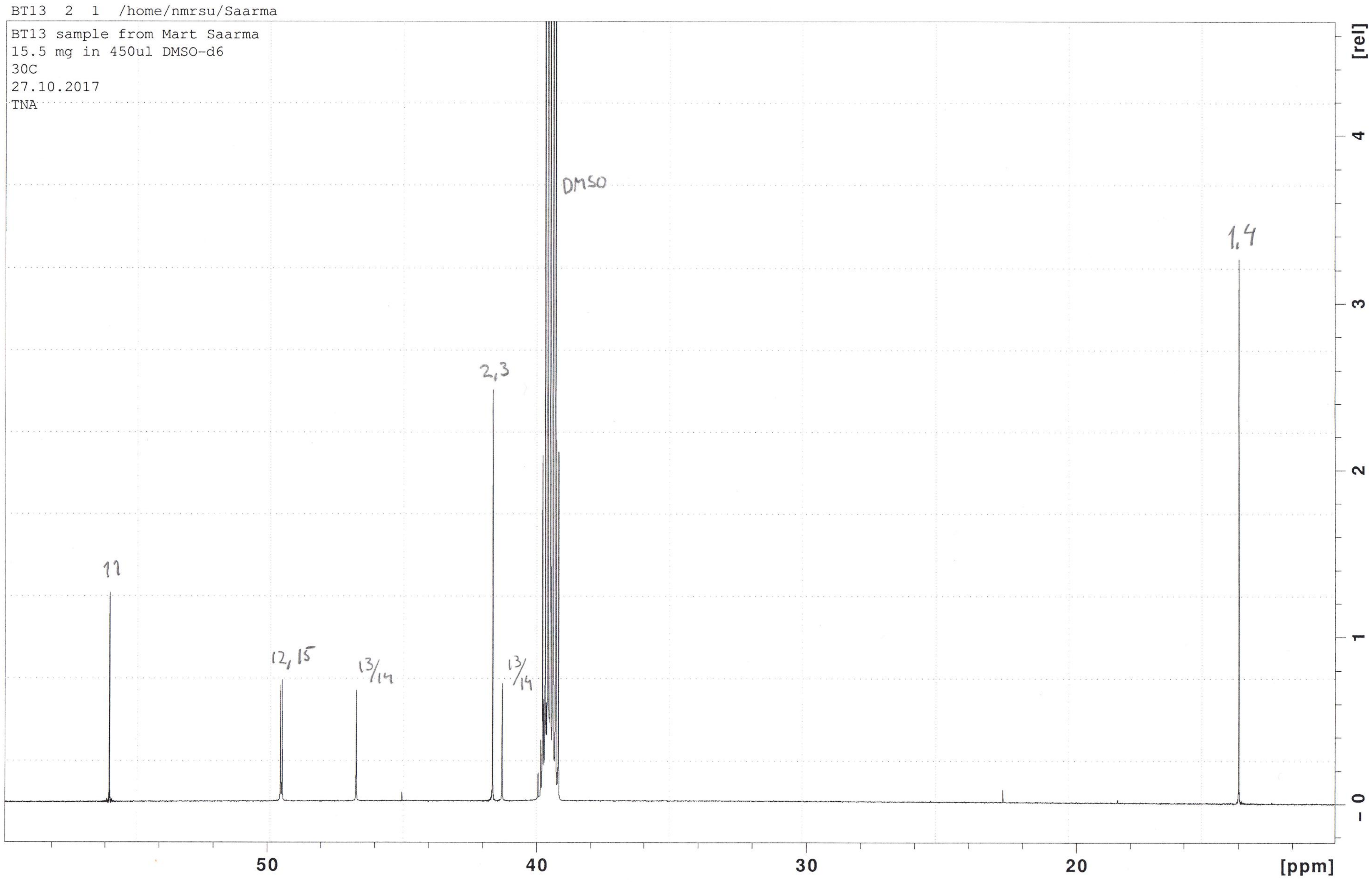
BT13 2 1 /home/nmrsu/Saarma
BT13 sample from Mart Saarma
15.5 mg in 450ul DMSO-d6
30C
27.10.2017
TNA
11
12,15
13/14
2,3
13/14
DMSO
1,4
50
40
30
20
[ppm]
0
1
2
3
4
[rel]

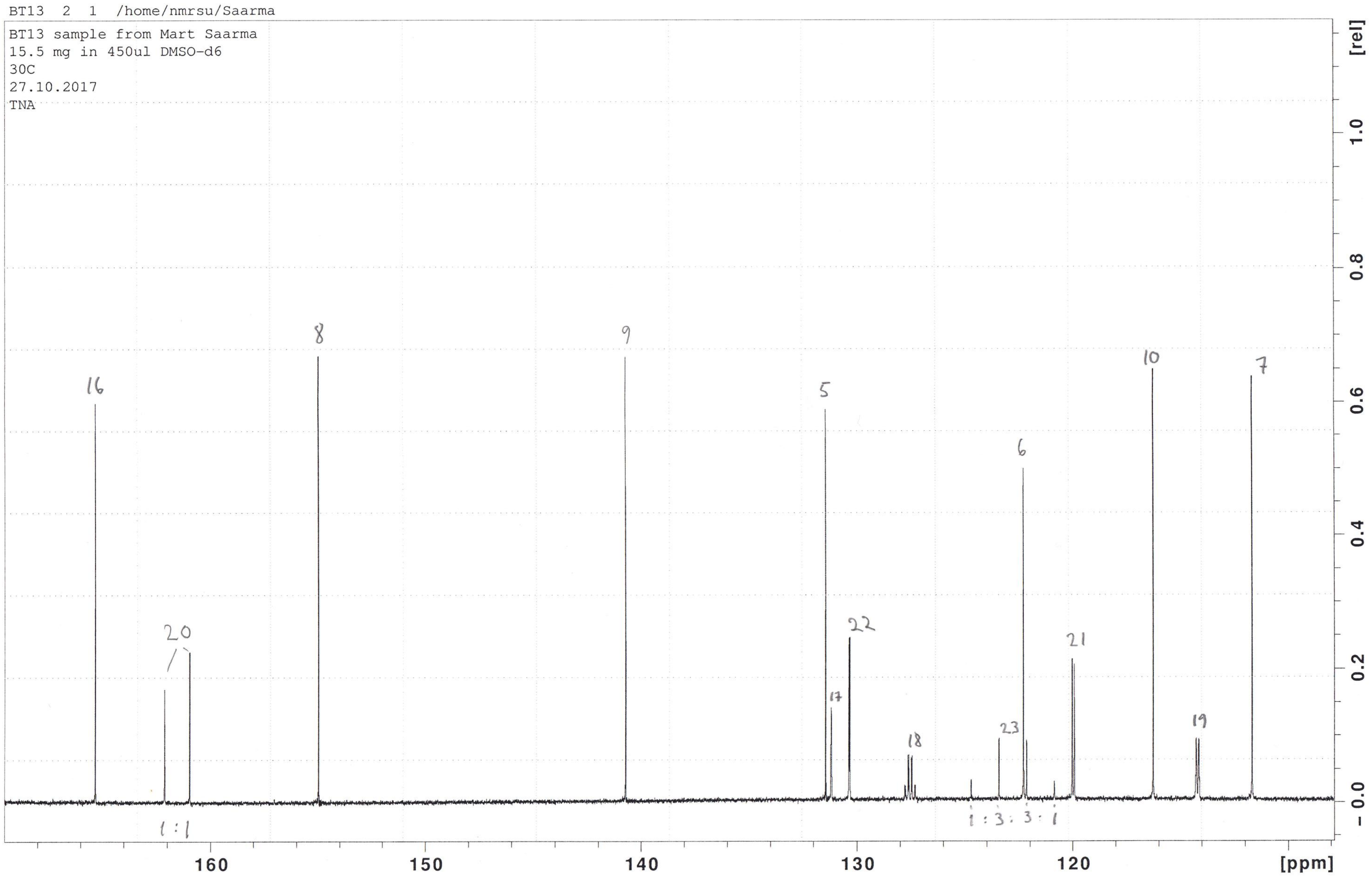
BT13 2 1 /home/nmrsu/Saarma
BT13 sample from Mart Saarma
15.5 mg in 450ul DMSO-d6
30C
27.10.2017
TNA
16
20
1 : 1
8
9
5
17
22
18
23
6
1 : 3 : 3 : 1
21
10
19
7
160
150
140
130
120
[ppm]
– 0.0
0.2
0.4
0.6
0.8
1.0
[rel]

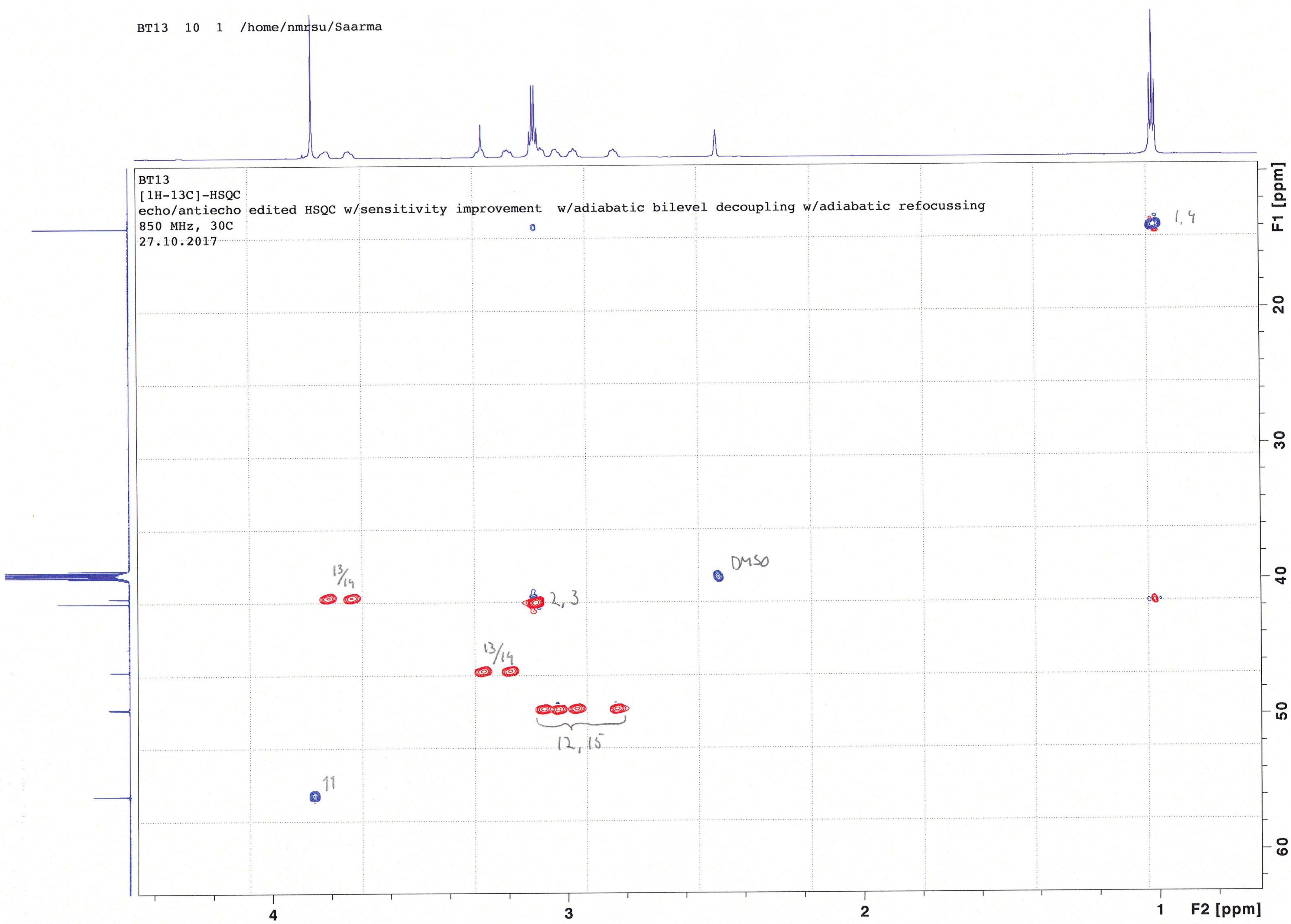
BT13 10 1 /home/nmrsu/Saarma
BT13
[1H-13C]-HSQC
echo/antiecho edited HSQC w/sensitivity improvement w/adiabatic bilevel decoupling w/adiabatic refocussing
850 MHz, 30C
27.10.2017
1,4
DMSO
13/14
2,3
13/14
12,15
11
4
3
2
1
F2 [ppm]
20
30
40
50
60
F1 [ppm]

BT13 10 1 /home/nmrsu/Saarma

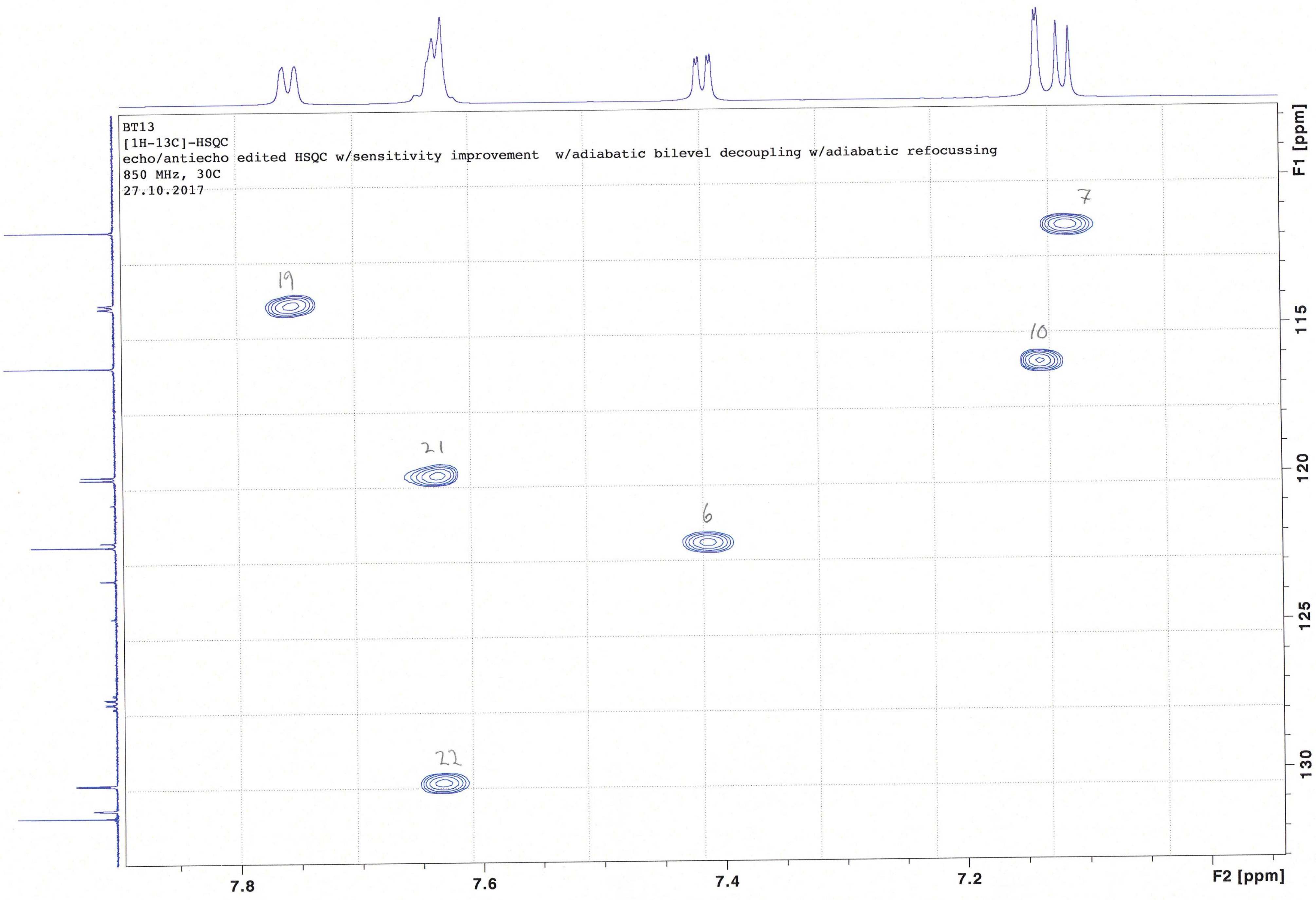

BT13 20 1 /home/nmrsu/Saarma

HMBC

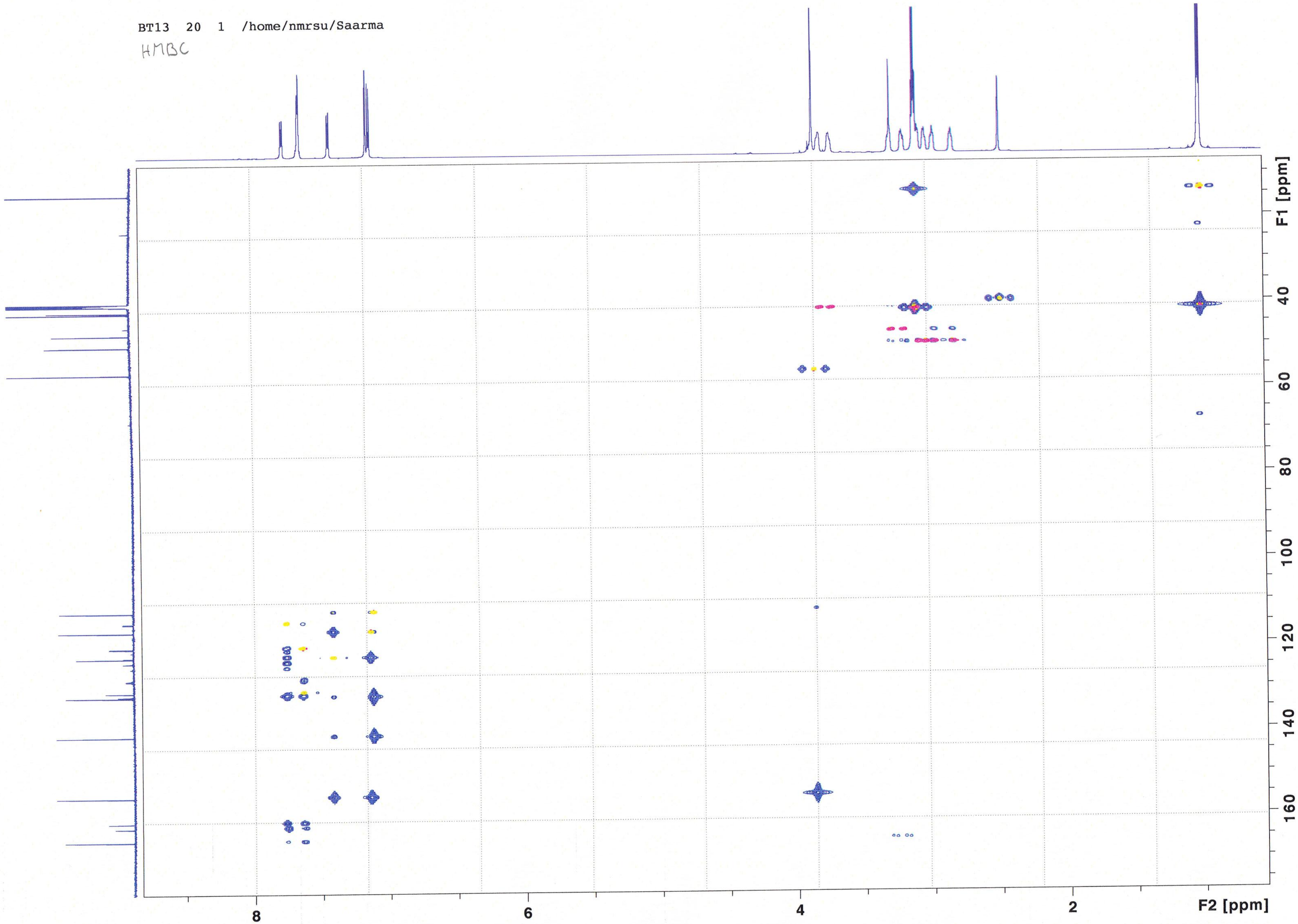

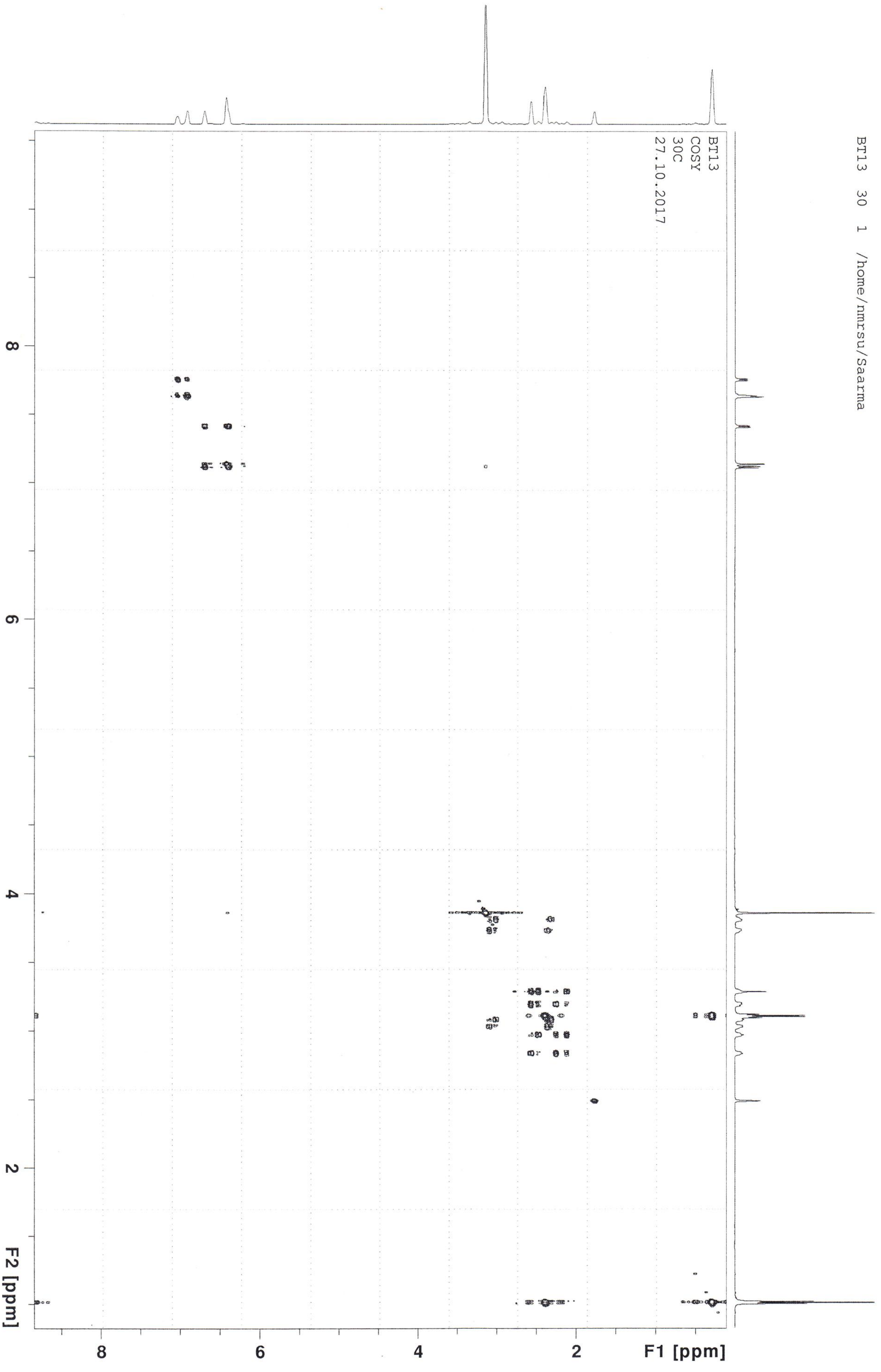
BT13 30 1 /home/nmrsu/Saarma
BT13
COSY
30C
27.10.2017
8
6
4
2
F2 [ppm]
8
6
4
2
F1 [ppm]

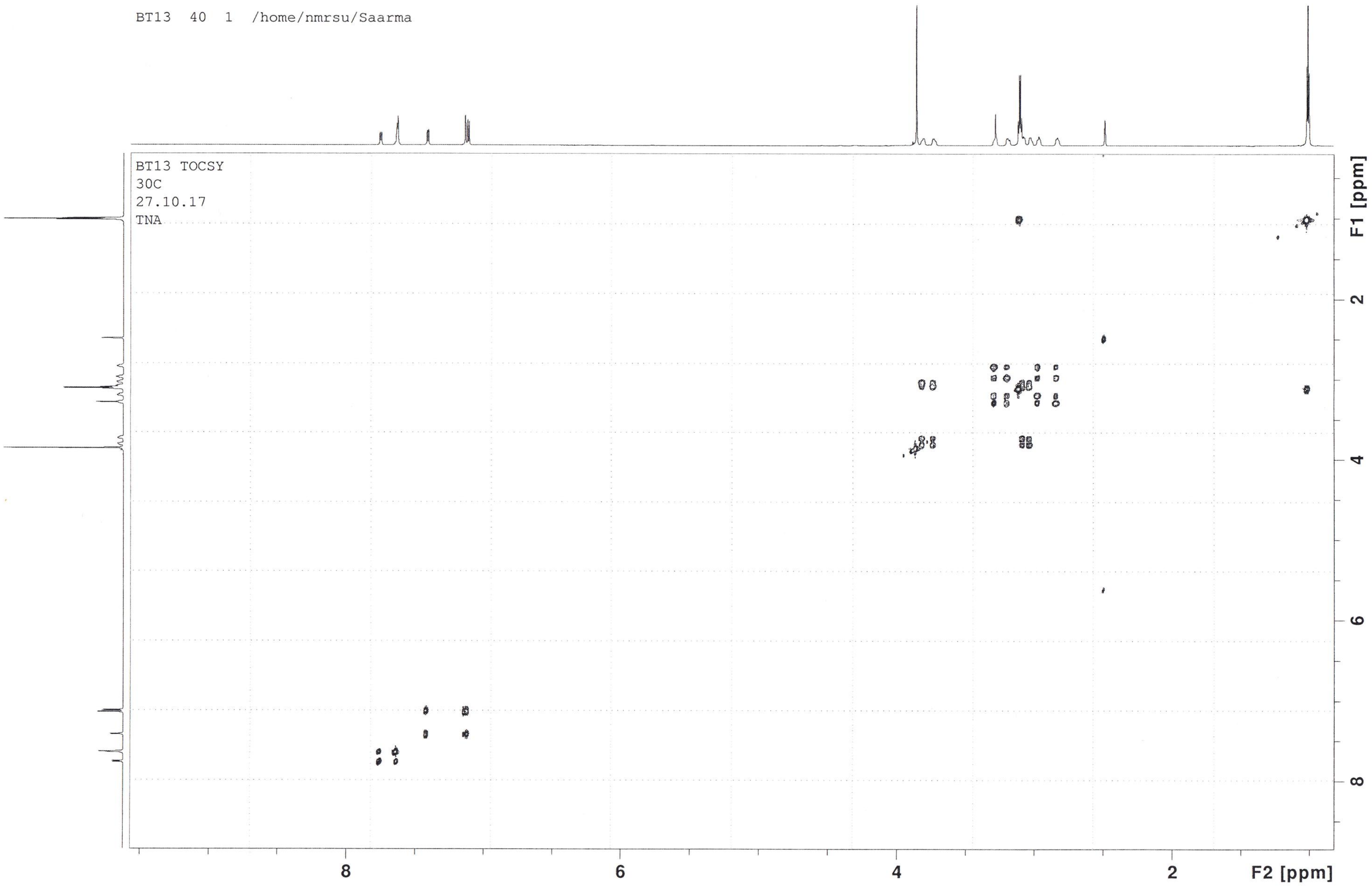
BT13 40 1 /home/nmrsu/Saarma
BT13 TOCSY
30C
27.10.17
TNA
F1 [ppm]
2
4
6
8
F2 [ppm]

Experimental animals:

Adult male mice C57Bl/6 (JRccHsd) (Harlan, the Netherlands) of 8–15 weeks of age weighing 19–32 grams were used for brain microdialysis experiments and for immunohistochemistry of phosphorylated extracellular-signal-regulated kinases 1 and 2 (pERK) and phosphorylated ribosomal protein S6 (pS6) in striatum. E13.5 embryos of NMRI mice and RET knock-out mice (C57BL/6JOlaHsd) (Laboratory Animal Centre, University of Helsinki) were used for primary cultures of midbrain dopamine neurons. C57Bl/6 mice were housed individually or in groups of 2–10 animals per cage. All animals were kept under 12:12 h light-dark cycle (lights on at 6 am) and ambient temperature in the animal facilities was 22 °C. Standard rodent chow (Harlan) and tap water were available *ad libitum*. All experiments were carried out according to the European Community guidelines for the use of experimental animals and approved by the National Animal Experiment Board of Finland (license numbers: ESAVI/7551/04.10.07/2013, ESAVI/198/04.10.07/2014) for experiments with living animals and the Laboratory Animal Centre of the University of Helsinki (license number: KEK15-022) for collection of E13.5 embryos of NMRI mice.

Genotyping

Mouse E13.5 embryos were genotyped using PCR for identification of embryos lacking RET expression as described previously (3). Tail snips from each embryo were collected in separate tubes and DNA was isolated using AccuStart II PCR Genotyping Kit (Quantabio, USA). Briefly, 100 µl of DNA extraction reagent was added to each tube with a tail snip. The samples were boiled at 95°C for 30 minutes. After cooling to room temperature, equal volume of stabilization buffer was added to the tube. PCR reaction mixture was prepared by adding 1 µl of DNA extract to mixture containing AccuStart II GEL Track PCR Supermix (2X), 400 nM primers P1-P4 (see below) and nuclease-free water. After the initial denaturation cycle (94°C, 2 minutes), DNA was amplified for 30 cycles in the following conditions: 94°C, 30 seconds; 60°C, 30 seconds; 72°C, 30 seconds. The sequences of the primers used in the reactions were: P1, 5′-TGGGAGAAGGCGAGTTTGGAAA-3′; P2, 5′-

TTCAGGAACACTGGCTACCATG-3′ for wild allele and P3, 5′-AGAGGCTATTCGGCTATGACTG-3′; P4, 5′-CCTGATCGACAAGACCGGCTTC-3′ for mutant allele.

Luciferase assay

Luciferase assay was performed as described earlier (2,4). Reporter cells (200 000 cells/ml) were plated to 96-well tissue culture plates (100 µl/well) a day before stimulation with BT13 or GDNF or FGF-2 in DMEM, 10% fetal bovine serum (FBS), 15 mM HEPES pH 7.2, 1% DMSO, 100 µg/ml normocin. The next day 100 µl of 2xGDNF or 2xBT13 solutions in DMEM, 15 mM HEPES, pH 7.2 and 1% DMSO were added to the reporter cells. The cells were cultured in the presence of the tested substances for 24 hours to produce luciferase. Luciferase activity was measured using neolite reagent (PerkinElmer, USA) according to the manufacturer's instruction. The luminescence was detected using FLUOstar OPTIMA microplate reader (BMG LABTECH). Compounds were tested in quadruplicates in 3 independent experiments.

Cell culture, cell transfection, treatments and sample preparation for phosphorylation assays

MG87RET cells were cultured overnight in 6-well plates and transfected with hGFRα1, hGFRα2 and GFP-expressing plasmid using Lipofectamine 2000 (Invitrogen), as described by the manufacturer. The next day, cells were starved for 4 hours using starvation medium (DMEM, 15 mM HEPES pH 7.2, 1% DMSO). The cells were stimulated by different concentrations of BT13 (10, 25, 50, 100 µM) and GDNF (250 ng/ml) or NRTN (250 ng/ml) dissolved in starvation medium for 15 minutes. Then the cells were washed with ice-cold PBS (phosphate buffered saline, pH 7.4; 137 mM NaCl, 2.7 mM KCl, 10 mM PO_4^{3-}) containing 1 mM Na_3VO_4 and lysed with 500µl RIPA-modified buffer (50 mM Tris-HCl, pH 7.4, 150 mM NaCl, 1 mM EDTA, 1% NP-40, 1% TX-100, 10% glycerol, EDTA-free protease inhibitor cocktail (Roche, Switzerland), 1 mM Na_3VO_4, 6 mM sodium deoxycholate, 1 mM PMSF) per well.

Western blotting-based ERK and AKT phosphorylation assay

Levels of pERK and pAKT in immortalized cells were analyzed as described previously (4). Cell lysate (100 µl) (see above) was collected and mixed with the same amount of 2xLaemmli buffer. Samples were boiled for 10 minutes and proteins were resolved on a 12% sodium dodecyl sulfate–polyacrylamide gel SDS-PAAG. After electrophoresis, proteins were electro-transferred onto nitrocellulose membranes. Lower part of the membranes containing ERK among others proteins was blocked with 10% skimmed milk in TBS-T (TBS (50 mM Tris-HCl, pH 7.4, 150 mM NaCl) with 0.15% of Tween 20) whereas the upper part containing AKT among other proteins was blocked with 10% BSA in TBS-T for 10 minutes and probed with E4 pERK (1:1000 in 3% skimmed milk in TBS-T; Santa Cruz Biotechnology Cat# sc-7383, RRID:AB_627545) and pAKT (1:500 in 3% BSA in TBS-T; Cell Signaling Technology Cat# 9271, RRID:AB_329825) antibodies, respectively, overnight at 4°C. The next day, both membranes were washed 3 times for 10 minutes with TBS-T. ERK-containing membrane was incubated with horseradish peroxidase (HRP)-conjugated anti-mouse secondary antibody (DAKO, Cat# P0447), diluted 1:3000 in 3% skimmed milk in TBS-T, and AKT-containing membrane was incubated with HRP-conjugated anti-rabbit antibody (GE Healthcare Cat# NA934, RRID:AB_772206), diluted 1:3000 in 3% BSA in TBS-T for 1 hour at room temperature. The membranes were washed again 4 times for 15 minutes with TBS-T. Bands were visualized with ECL Plus Western Blotting Substrate (Pierce, USA) or SuperSignal West Femto Maximum Sensitivity Substrate (Pierce) using Luminescent Image Analyzer LAS3000 (Fuji-Film, Japan). Membranes were stripped and re-probed with GAPDH antibody (1:4000, Millipore Cat# MAB374, RRID:AB_2107445) for 1 hour at room temperature to confirm equal loading of the proteins to the gels. Membranes were washed and incubated with HRP-conjugated anti-mouse secondary antibody for 1 hour and visualized according to the above-mentioned procedure.

Analysis of RET phosphorylation by Western blotting

The level of RET phosphorylation in the cells was analyzed by Western blotting as described previously (5). Cell lysates prepared as described above were centrifuged (5000 rpm, 5 min, 4ºC) to precipitate cell debris. RET was immunoprecipitated from the supernatant with 2 µg/ml anti-RET C-20 antibody (Santa Cruz Biotechnology Cat# sc-1290, RRID:AB_631316) and Dynabeads Protein G (Thermo Fisher Scientific) overnight at 4ºC. The next day, the beads were washed 3 times with TBS with 1% Triton X-100. Immunoprecipitated proteins were eluted by adding 50 µl of 2xLaemmli buffer, resolved on 7.5% SDS-PAAG and transferred to nitrocellulose membrane. The membrane was blocked with 10% skimmed milk in TBS-T for 10 minutes and probed overnight with phospho-tyrosine-specific antibody (1:1500 in 3% skimmed milk in TBS-T; Merck Millipore Cat# 05-321, RRID:AB_309678). The membrane was washed 3 times for 10 minutes with TBS-T and incubated with HRP-conjugated anti-mouse secondary antibody (1:3000 in 3% skimmed milk in TBS-T) for 1 hour at room temperature. The membrane was washed again with TBS-T 4 times for 15 minutes. Bands were visualized with with ECL Plus Western Blotting Substrate or SuperSignal West Femto Maximum Sensitivity Substrate using Luminescent Image Analyzer LAS3000. Loading control was confirmed by re-probing the membrane with anti-RET C-20 antibody (1:500) for 1 hour at room temperature after stripping. The membrane was washed and incubated with HRP-conjugated anti-goat secondary antibody (1:1500, DAKO, Cat# P0449) for 1 hour and visualized according to the above-mentioned procedure.

RET-ELISA assay

To quantify the level of phosphorylated RET in immortalized cells we performed phospho-RET-ELISA assays (6). Assay plates (OptiPlate 96 F HB, Wallac) were coated with anti-RET C-20 antibody (1 µg/ml in TBS overnight), washed 4 times with PBS, blocked with blocking solution (5% BSA in TBS), 3 hours at room temperature and then washed with RIPA modified buffer. Cell lysates prepared as described above were added to the plates (70 µl/well) and incubated overnight at 4°C on

a shaker. The plates were washed 3 times with washing buffer (TBS, 1% Triton X-100, 2% glycerol) and probed with phospho-tyrosine-specific antibody (1:1000 in binding buffer (TBS, 1% Triton X-100, 2% glycerol, 2%BSA)) for 1 hour at room temperature. The plates were washed 3 times with washing buffer, incubated with HRP-conjugated anti-mouse secondary antibody (1:3000 in binding buffer) for 1 hour at room temperature and washed once more 3 times with washing buffer. To detect the signal, pre-warmed SuperSignal West Femto Maximum Sensitivity Substrate (100 μl/well) was applied to the plates and luminescence was counted using FLUOstar OPTIMA microplate reader (BMG LABTECH).

Survival assay for naïve dopamine neurons

Plate preparation

Plates were prepared as described previously (7) with small modifications. A 96-well plate was pre-coated with poly-DL-ornithine, (0.5 mg/ml in 0.15 M borate buffer, pH 8.7, Sigma-Aldrich, Cat# P8638), overnight at 4ºC. The plate was washed 3 times with PBS and a micro-island was created in each well by circling at the edge of the well with the tip of a suction pump. The plate was dried for 1 hour in laminar flow hood and stored at 4°C before use.

Preparation of primary neuronal cultures from mouse embryonic midbrain

Neuronal cultures were prepared from E13.5 embryos of NMRI mice (7,8). The midbrain floors were dissected in Dulbecco's medium (0.1 g/l $MgCl_2$•$6H_2O$, 0.1 g/l $CaCl_2$, 8 g/l NaCl, 0.2 g/l KCl, 1.4 g/l Na_2HPO_4•$2H_2O$, 0.2 g/l KH_2PO_4) containing 2% BSA under a dissection microscope (Olympus SZX10 Stereo Microscope), washed 3 times with calcium and magnesium-free Hank's Balanced Salt Solution (HBSS: Gibco, Life Technologies) and incubated in 5 mg/ml trypsin solution in HBSS for 20 minutes at 37°C. Enzymatic activity of trypsin was blocked by adding equal volume of FBS, containing 0.1 mg/ml of DNase I (Roche, Cat# 11284932001). Cells were triturated with a siliconized glass Pasteur pipette to get single cell suspensions and centrifuged at 1000 rpm for 5 minutes. The pellets were washed 3 times with primary neuron culture medium [(Dulbecco's MEM/Nut mix F12

(Invitrogen/Gibco, Cat# 21331-020), 1xN_2 serum supplement (Invitrogen/Gibco, Cat# 17502-048), 33 mM D-glucose (Cat # G-8769, Sigma-Aldrich), 0.5 mM L-glutamine (Invitrogen/Gibco, Cat# 25030-032), and 100 µg/ml Primocin (InvivoGen, Cat# ant-pm-2)] to remove the traces of serum. The washed pellets were resuspended in 150–200 µl of the primary neuron culture medium. The cells were counted using a TC20 automated cell counter (BIO-RAD). About 30000 cells were plated per well of previously prepared plates and cultured at 37°C.

Different concentrations of BT13 (0.01, 0.1, 1, 10 µM) and GDNF (Icosagen, 10 ng/ml) were dissolved in primary neuron culture medium containing 1% DMSO and applied to the wells within 1-hour post plating. The cells were incubated for 5 days and half of the culture media were replaced with fresh portions 2.5 days post plating.

Tyrosine hydroxylase and Gamma-Aminobutyric Acid immunocytochemistry

Dopamine neurons were visualized by immunocytochemical staining with an antibody against tyrosine hydroxylase (TH), the key enzyme of dopamine synthesis. After 5 days of culturing, cells were fixed with 4% paraformaldehyde (PFA) for 20 minutes and washed 3 times with PBS followed by permeabilization with 0.2% Triton X-100 in PBS for 15 minutes. Unspecific binding was blocked by incubating cells with a blocking solution (5% horse serum in 0.2% Triton X-100 in PBS) for 1 hour. Anti-TH antibody (1:500 in blocking solution, Millipore Cat# MAB318, RRID:AB_2201528) and anti-γ-aminobutyric acid (GABA) antibody (Catalogue number: ABN131 Chemicon/Merck., diluted 1:500 in blocking solution) was applied to the cells and incubated overnight at 4°C. Cells were washed 3 times with PBS and incubated with Alexa Fluor 647 conjugated anti-mouse or anti-rabbit secondary antibody (1:500 in blocking solution, Thermo Fisher Scientific Cat# A-31571, RRID:AB_162542) for 1 hour at room temperature. Cells were washed with PBS to remove unbound antibody and nuclei were stained with 0.2 µg/ml DAPI (4', 6-diamidino-2-phenylindole) in PBS for 10 minutes at room temperature. Finally, cells were washed 3 times with PBS and kept in PBS until imaging.

Cells were imaged by CellInsight (CX51110, ThermoFisher Scientific) CX5 High Content Screening (HCS) with 20× magnification. Images were analyzed using CellProfiler image analysis software (9).

Survival of 6-OHDA and MPP^+ challenged dopamine neurons

The survival of 6-OHDA and MPP+ challenged dopamine neurons was performed using previously described method with modifications (10)[DOI:10.1021/acschemneuro.9b00396]. The dopamine neurons were cultured for 5 days in dopamine cell culture media [Dulbecco's MEM/Nut mix F12 (Invitrogen/Gibco; 21331–020), 1xN2 serum supplement (Invitrogen/ Gibco; 17502–048), 33mM D-Glucose (Sigma; G-8769), 0.5 mM L-Glutamine (Invitrogen/ Gibco; 25030–032), and 100 µg/ml Primocin (Invivo Gen)]. The culture procedure was similar to that of naïve dopamine neurons culture as described above. On 6th day in vitro (DIV), the cultured neurons were challenged with 30 µM of 6-OHDA for 72 hours or 2 µM of MPP+ for 48 hours. BT13 (0.1 and 1 µM) and GDNF (10 ng/ml) were applied simultaneously with toxin. All the solutions were prepared in 1% DMSO containing cell culture media. The cells were fixed and probed with antibody against tyrosine hydroxylase (TH, key enzyme of dopamine synthesis) overnight at 4°C. Cells were washed 3 times with PBS and incubated with Alexa Fluor 647 conjugated anti-mouse secondary antibody (1:500 in blocking solution, Thermo Fisher Scientific Cat# A-31571, RRID:AB_162542) for 1 hour at room temperature. Cells were washed with PBS to remove unbound antibody and nuclei were stained with 0.2 µg/ml DAPI (4', 6-diamidino-2-phenylindole) in PBS for 10 minutes at room temperature. Finally, cells were washed 3 times with PBS and kept in PBS until imaging. Cells were imaged by ImageXpress Nano Automated Imaging System (Molecular Devices) 10×magnification. Images were analyzed using CellProfiler image analysis software (9).

The survival analysis of MPP+ challenged wild-type dopamine neurons was also performed by Neuron Experts company (http://www.neuronexperts.com/) using previously described method (11). Ventral portion of the mesencephalic flexure was dissected from the brains of rat E15 embryos and trypsinized for 20 minutes at 37°C in 1% trypsin-EDTA (Invitrogen) diluted in Ca^{2+} and Mg^{2+}-free

PBS. The reaction was stopped by addition of DMEM containing 0.1 mg/ml DNaase I (Roche) and 10% FBS. Cells were dissociated by trituration, precipitated by centrifugation, plated on poly-L-lysine precoated 96-well plates (69,000 cells/well) and cultured in Neurobasal medium (Invitrogen) supplemented with 2% B27 (Invitrogen), 0.2 mM L-glutamine and 1% Penicillin-Streptomycin. Half of the culture medium was changed every two days with fresh medium. On 6th day in vitro (6th DIV) the culture medium was replaced by the fresh media containing 16 μM MPP^+ and tested substances or DMSO as a negative control. Brain-derived neurotrophic factor (BDNF) (10 ng/ml) that is known to promote the survival of cultured dopamine neurons (12) was used as a positive control. After 48 hours (8th DIV) cells were fixed with 4% PFA. Dopamine neurons were labelled using antibody against TH (Sigma-Aldrich Cat# T1299, RRID:AB_477560) and Alexa Fluor 488 conjugated goat anti-mouse antibody (Molecular Probes Cat# A-11017, RRID:AB_143160). Nuclei were labelled with Hoechst dye. For each condition, 2x10 pictures per well were taken using InCell AnalyzerTM 1000 (Amersham Biosciences, UK) with 10x magnification. All images were taken using the same conditions. The number of TH-positive neurons was analysed using InCell AnalyzerTM 1000 3.2.Workstation software.

Survival of MPP^+ challenged dopamine neurons after CRISPR/Cas9-mediated deletion of RET receptor

Dopamine neurons were cultured as described above. Lentiviral vectors LentiCRISPR/Cas9 with guide RNA targeting mouse RET or LentiCRISPR/Cas9 with scrambled guide RNA were produced and added to the neuronal cultures one hour post-plating as described in (13). On 8th DIV, the cells were treated with MPP+ (2 μM), BT13 (0.1 and 1 μM) or GDNF (10 ng/ml) for 48 hours. The cells were fixed and immunostained with antibody against TH overnight at 4°C. Cells were washed 3 times with PBS and incubated with Alexa Fluor 647 conjugated anti-mouse secondary antibody for 1 hour at room temperature. Cells were washed with PBS and nuclei were stained with 0.2 μg/ml DAPI in PBS for 10 minutes at room temperature. Cells were imaged by ImageXpress Nano Automated

Imaging System (Molecular Devices) at 10× magnification. Images were analyzed using CellProfiler/Cell Analyst image analysis software.

Analysis of pERK, pS6 and pAKT levels in the cultured dopamine neurons treated with BT13 and GDNF

Dopamine neurons were cultured as described above with slight modifications. The cells were prepared and cultured on cover-slip coated with poly-DL-ornithine for 48-hours. The cells were starved for 4-hours using starvation media [(Dulbecco's MEM/Nut mix F12 (Invitrogen/Gibco, Cat# 21331-020;1% DMSO]. After starvation, dopamine neurons were treated with 1 μM of BT13 and 10 ng/ml of GDNF for 5 minutes for phospho (p)ERK activation and 1 hour for phospho (p)Akt activation (treatment time was chosen based on the data from pilot experiment). All the solutions were prepared in starvation media. After treatment for relevant time point, the cells were fixed and probed with Anti-TH antibody (1:500, Millipore Cat# MAB318, RRID:AB_2201528) , pERK (1:500, Cell Signaling, Cat# 4370L RRID:AB_2297462), phospho-ribosomal protein S6 (pS6, downstream target of AKT) (1:500, Cell Signaling, Cat# 4858L RRID:AB_1031194) and pAKT (1:500, Cell Signaling, Cat# 3787L RRID:AB_331170) antibodies in blocking solution overnight at 4°C (4,14). Cells were washed 3 times with PBS and incubated with Alexa Fluor 488 conjugated anti-mouse (Thermo Fisher Scientific Cat# A-21202, RRID: AB_141607) and Alexa Fluor 647 conjugated anti-rabbit secondary antibody (Thermo Fisher Scientific Cat# A-31573, RRID: AB_141607) diluted 1:500 in blocking solution for 1 hour at room temperature. Cells were washed with PBS to remove unbound antibody and nuclei were stained with 0.2 μg/ml DAPI (4', 6-diamidino-2-phenylindole) in PBS for 10 minutes at room temperature. Finally, cells were washed 3 times with PBS and cover-slips were mounted to glass slide with Immu-mount (Thermo Fisher Scientific, Cat# 9990402).

Cells were scanned by 3DHISTECH Pannoramic 250 FLASH II digital slide scanner with 40×magnification. The mean intensity of pERK and pS6-specific staining in TH-positive cells was

measured using ImageJ software (Media Cybernetics Inc, USA) and normalized to the area of dopamine neuron where intensity was measured.

Analysis of pERK and pS6 levels in the mouse brains treated with BT13 and GDNF.

Microinjections and stereotaxic surgery

Bilateral microinjections into the mouse dorsal striatum (AP = +0.6; ML = +/-1.8; DV = -2.2 relative to the bregma, according to the mouse brain atlas (15) were performed using stereotaxic frame (Stoelting, USA). The stereotaxic surgery was conducted under isoflurane (Vetflurane 1000 mg/g, Virbac) anaesthesia. The animals received buprenorphine 0.1 mg/kg s.c. (Temgesic® 0.3 mg/ml, Indivior UK Limited) for analgesia. A small amount of lidocaine-adrenalin-solution (10 mg/ml; Orion Pharma) was injected between the skull and the scalp for local anaesthesia and to prevent bleeding. Tested substances were delivered into the striata using an electronic injector (Quintessential stereotactic injector, Stoelting, USA) with a 10 µl microsyringe (World Precision Instruments, United Kingdom). Injection speed was set to 0.2 µl/min and injection volume to 2 µl. Left striatum received BT13 or GDNF dissolved in saline with 0.5 % DMSO (vehicle). The right striatum was always injected with the vehicle. BT13 was injected in the doses of 103.5 µg (≈100 µg) (N = 4), 207 µg (≈200 µg) (N = 4), 517.5 µg (≈500 µg) (N = 4) and 776.25 µg (≈750 µg) (N = 4) and GDNF in the dose of 5 or 10 µg (N = 4). At the completion of the injection, the needle was kept in place for 4 minutes and then slowly withdrawn to minimize backflow of the solution. The animals were allowed to recover in their home cage on a heating pad for 1 hour after the injection. Afterwards the mice were anesthetized with sodium pentobarbital (100 mg/kg, i.p.; Mebunat Vet 60 mg/ml, Orion Pharma) and transcardially perfused with warm PBS for 4 minutes followed by warm (temperature: 25-35^{0}C) 4% PFA in 0.1 M phosphate buffer (pH 7.4) for 7 minutes. The brains were removed and postfixed in 4% PFA overnight at room temperature.

Immunohistochemistry

Immunohistochemical staining of pERK1/2 and pS6 were performed on 5 μm thick coronal sections of paraffin-embedded mouse brains using primary antibodies raised against pERK1/2 (1:300, Cell Signaling Technology Cat# 4370, RRID:AB_2315112) and pS6 (1:300, Cell Signaling Technology Cat# 5364, RRID:AB_10694233). To visualize the stainings, sections were incubated with HRP-conjugated goat anti-rabbit secondary antibody (1:500, Sigma-Aldrich Cat# A6154, RRID:AB_258284), washed 3 times in TBS and treated with 3,3´-diaminobenzidine (DAB; Cat# SK-4100, USA) as a chromogen.

Stained sections were dehydrated in series of ethanol solutions with increasing concentrations, clarified in xylen and finally mounted in DePeX® mounting medium (VWR International Ltd., England). In all cases endogeneous peroxidase activity was quenched by preincubation of sections with 3% H_2O_2 solution.

Analysis of pERK, pS6 and pAKT immunohistochemistry

The stained sections were scanned using an automated bright field microscopy slide scanner (3DHistech Ltd., Hungary). Mean optical density (OD) of the staining in a single section (closest to the injection site and with the highest signal) from both hemispheres of each animal was analyzed using ImageJ software (Media Cybernetics Inc, USA). In cases where the highest signal was not located in the same section for both hemispheres, the values were counted from separate sections.

Digital images of the sections were first converted into 8-bit grey scale and inverted. Then roughly equal sized areas were outlined from both hemispheres and OD values were counted within these areas. Background OD values were measured from the peripheral striatal area that lacked the signal or from the septum and subtracted from the OD values in the areas selected for analysis. Resulting data were normalized to the area of analysed selections and subjected to the statistical analysis. For presentation purpose the values for BT13- and GDNF-treated sides were normalized to the values for vehicle-treated side of the same brain.

In vivo microdialysis

For *in vivo* microdialysis 2 months old C57BL/6J male mice were used. Each animal underwent 2 dialyses with 2 days washout period between them. A microdialysis guide cannula (MAB 4.1, AgnTho's, AB, Sweden) was inserted into the dorsal striatum (AP = +0.6; ML = +1.8; DV = -2.2 relative to the bregma, according to the mouse brain atlas (15) under isoflurane (Vetflurane 1000 mg/g, Virbac, France) anaesthesia and attached to the skull by two stainless steel screws and dental cement (Aqualox; Voco, Germany). Lidocaine-adrenalin-solution (10 mg/ml; Orion Pharma, Finland) was injected between the skull and the scalp for local anaesthesia and to prevent bleeding. Buprenorphine 0.1 mg/kg s.c. (Temgesic® 0.3 mg/ml, Indivior UK Limited, United Kingdom) was administered before and 12 hours after the surgery for analgesia. After at least 4 days of recovery, a microdialysis probe (MAB 4.9.1.Cu; AgnTho's AB) was inserted into the guide cannula and dialysis was started with Ringer solution (147 mM NaCl, 1.2 mM $CaCl_2$, 2.7 mM KCl, 1.0 mM $MgCl_2$, and 0.04 mM ascorbic acid) at a flow rate of 2 µl/min. Sample collection started after 2 hours of stabilization at 15 minute intervals. BT13 from 10 mM DMSO-stock was dissolved in Ringer solution until desirable concentration (the highest tested concentration (50 µM) was limited by BT13 solubility). The solutions were sonicated to enhance solubility of BT13. The final solutions contained less than 0.5% of DMSO, which alone did not significantly influence extracellular dopamine levels (data not shown). Concentration of dopamine was analyzed with high-performance liquid chromatography (HPLC) using electrochemical detection (Coulochem II; ESA, Inc., USA). The column (Kinetex 2.6u; XB-C18; 50 x 4.6 mm; Phenomenex; USA) was kept at 45°C with a column heater. The flow rate of mobile phase (0.1 M NaH_2PO_4, pH 4.0, 0.1 mg/ml octanesulphonic acid, 1.0 mM EDTA and 8% methanol) was 1 ml/min. An autoinjector (SIL-20AC, Shimadzu, Japan) was used to inject 25 µl of the sample into the chromatographic system. After achieving a stable baseline (determined as an average of 4 consecutive samples) with Ringer solution, BT13 was delivered into the striatum as a continuous infusion by reverse dialysis until the end of the experiment.

Pharmacokinetics of BT13 and its effects on the levels of dopamine metabolites in the midbrain

Male Sprague Dawley rats were divided in to four groups (Vehicle, 0.5 hour, 1 hour and 2 hour) and dosed intravenously with vehicle (3% DMSO 40% PEG in 20 % HPCD water) or BT13 at the dose 10 mg/kg. Blood was collected by cardiac puncture for determination of BT13 concentrations in plasma. Brains were dissected out for determination of BT13 concentrations and neurochemical analysis in discrete brain regions.

Plasma samples were prepared for analysis by protein precipitation with acetonitrile containing internal standard (tolbutamide), centrifugation, and transfer of the resulting supernatant to a 96-well plate. Brain samples were homogenised 1:1 (w:v) in water, and then prepared for analysis in the same way as the plasma samples. Calibration standards consisted of control plasma spiked with analyte over the range 1-5000 ng/ml, control brain homogenate spiked with analyte over the range 1-5000 ng/g. These were prepared and analysed in the same way as the samples. Analysis of plasma and brain samples was carried out using Shimadzu Nexera X2 UHPLC/Shimadzu LCMS 8060/ Acquity BEH Phenyl (50 x 2.1) mm, 1.7 µm column). Gradient elution was employed, using acetonitrile and water, both acidified with 0.1% formic acid. Data were processed with Lab solutions software.

For neurochemical analysis midbrain samples were homogenised in 10 µl/mg of 0.4 M PCA buffer containing 0.05 mM EDTA and 1% $Na_2S_2O_5$ using a tissue lyser. Samples were spun at 4°C, 10,000 g for 10 min and the supernatant filtered using a 0.22 µm cellulose acetate filter (Spin-X). 10 µl of the filtered supernatant were injected into an HPLC system consisting on a CTC PAL autosampler, a Thermo 3000 HPLC pump and a Colouchem III detector. Dopamine (DA), 3,4-Dihydroxyphenylacetic acid (DOPAC) and Homovanillic acid (HVA) were separated using a C-18 reverse phase column, identified by retention time and quantified using calibration standards (1-1000 nM).

Experimental Design and Statistical Analysis

Experiments in cultured cells were repeated 3-8 times. The number of animals per group in *in vivo* pERK/pS6 analysis and microdialysis was selected on the basis of the previous experience (16) and is indicated in the description of each experiment. The data were subjected to statistical analysis using Student's t-test or one-way ANOVA with a Dunnett's *post hoc* tests in GraphPad Prism 6 (GraphPad Software Inc., USA). Results were considered statistically significant when P-value was lower than 0.05. Results are presented as Mean±SEM.

Supplementary Results

BT13 does not support the survival of gamma-aminobutyric acid (GABA) neurons

We also assessed the survival effect of BT13 on GABA neurons in mouse embryonic midbrain primary cultures. BT13 failed to influence the survival of cultured GABA neurons (Fig. S2).

BT13 stimulates intracellular signaling (AKT) important for neuronal survival and regeneration in the cultured dopamine neurons

We also assessed the ability of BT13 to activate AKT intracellular signaling cascades in cultured dopamine neurons by measuring the mean intensity of phosphorylated AKT (pAKT). BT13 was able to activate AKT (P=0.0063) in cultured dopamine neurons. BT13 increased the mean intensity of pAKT (Fig. S3) by 1.1 fold (1 μM, P= 0.0362) and GDNF (10 ng/ml) by 1.1 fold (P=0.0210); one-way ANOVA with Dunnett's *post hoc* test for all comparisons. Repeated twice

BT13 penetrates BBB and increases the levels of dopamine metabolites in the midbrain

We assessed BT13 concentration in plasma and different brain regions (frontal cortex, striatum, midbrain and cerebellum) 30 minutes, 1 hour and 2 hours post-intravenous administration. The data are presented as mean from 6 animals per group and in percentage from plasma concentration (Supplementary Table 1).

The dopamine, HVA and DOPAC concentrations in midbrain were measured 30 minutes post-intravenous administration. The measurement time point was was selected based on pharmacokinetics data for BT13. BT13 significantly increased the level of HVA by 2.4-fold (P=0.0488, unpaired t-test).

We also observed trends to increase in DA and DOPAC concentrations. The DOPAC/DA ratio remained unchanged, but HVA/DA ratio was slightly higher in the brain of animals treated with BT13 (Supplementary Fig. 3)

Supplementary Figures

Figure S1: BT13 does not support the survival of GABA neurons. The number of GABA-positive cells in the wild-type midbrain cultures on 5th DIV normalized to the total number of cells in the culture and presented as percentage of vehicle treated samples, average from 6 wells. The experiments were repeated 3 times with reproducible results. Concentration of GDNF is provided in ng/ml. VEH - Vehicle. RM ANOVA with Dunnett's *post hoc* test, Mean ± SEM, Number of wells (N) = 6.

Figure S2: BT13 activates intracellular signaling cascades (Akt) responsible for the survival and regeneration of neurons *in vitro.* Mean intensity of pAkt immunostaining of dopamine neurons in different treatment groups presented as percentage of vehicle-treated dopamine neurons. GDNF concentration: 10 ng/ml, BT13: 1 μM VEH - Vehicle. * $P < 0.05$, one-way ANOVA with Dunnett's *post hoc* test, Mean ± SEM, Number of independent experiments (N)=3

Figure S3: BT13 increases the level of dopamine metabolites in midbrain 30 min post intravenous injection. Concentration of dopamine and its metabolites is presented as nmol per gram of tissue sample at 30 minutes time point. VEH-Vehicle, * $P < 0.05$, Unpaired t-test, Mean ± SEM, Number of animals (N) = 6 in each group

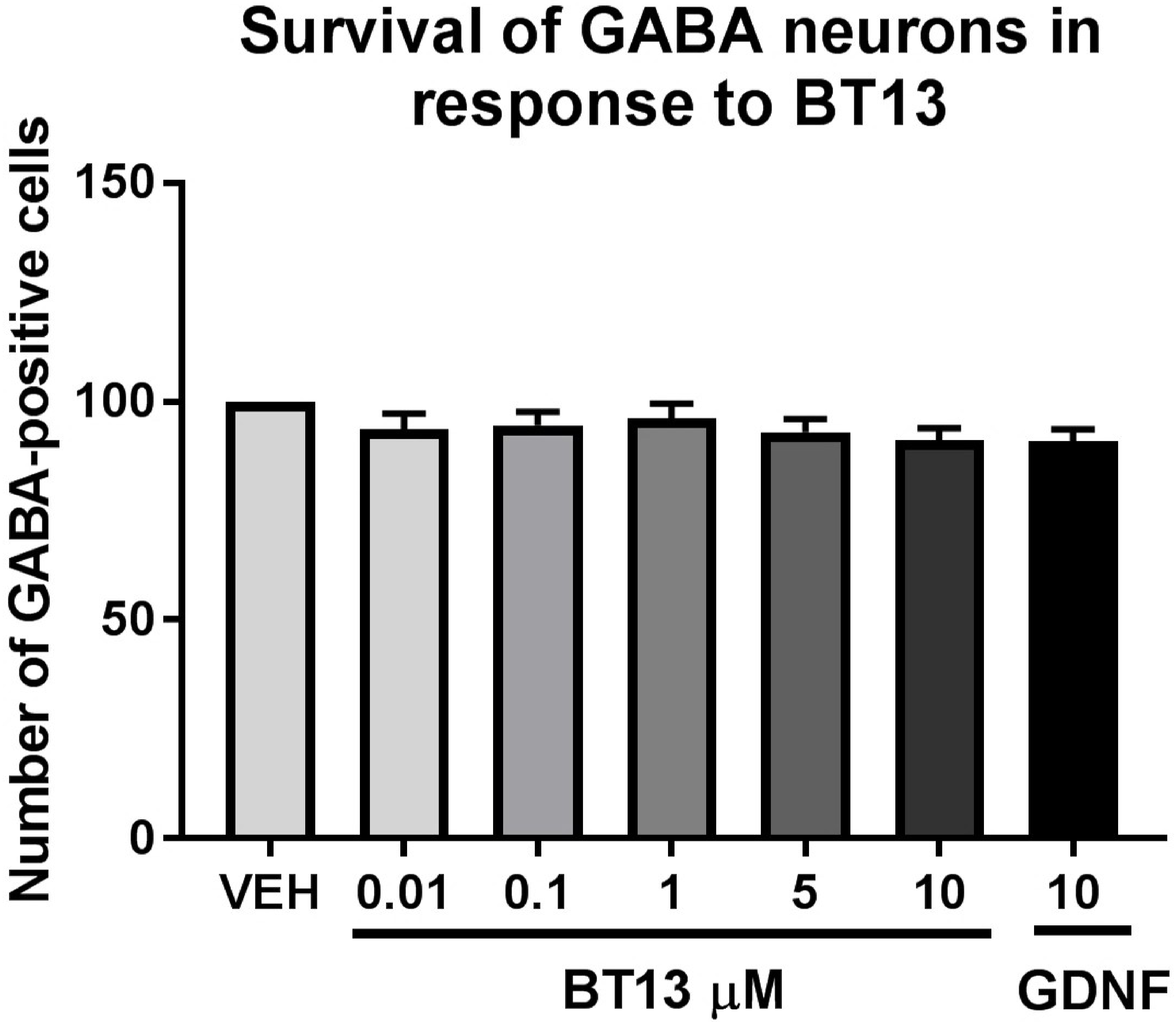
Survival of GABA neurons in response to BT13
Number of GABA-positive cells
150
100
50
0
VEH
0.01
0.1
1
5
10
10
BT13 μM
GDNF

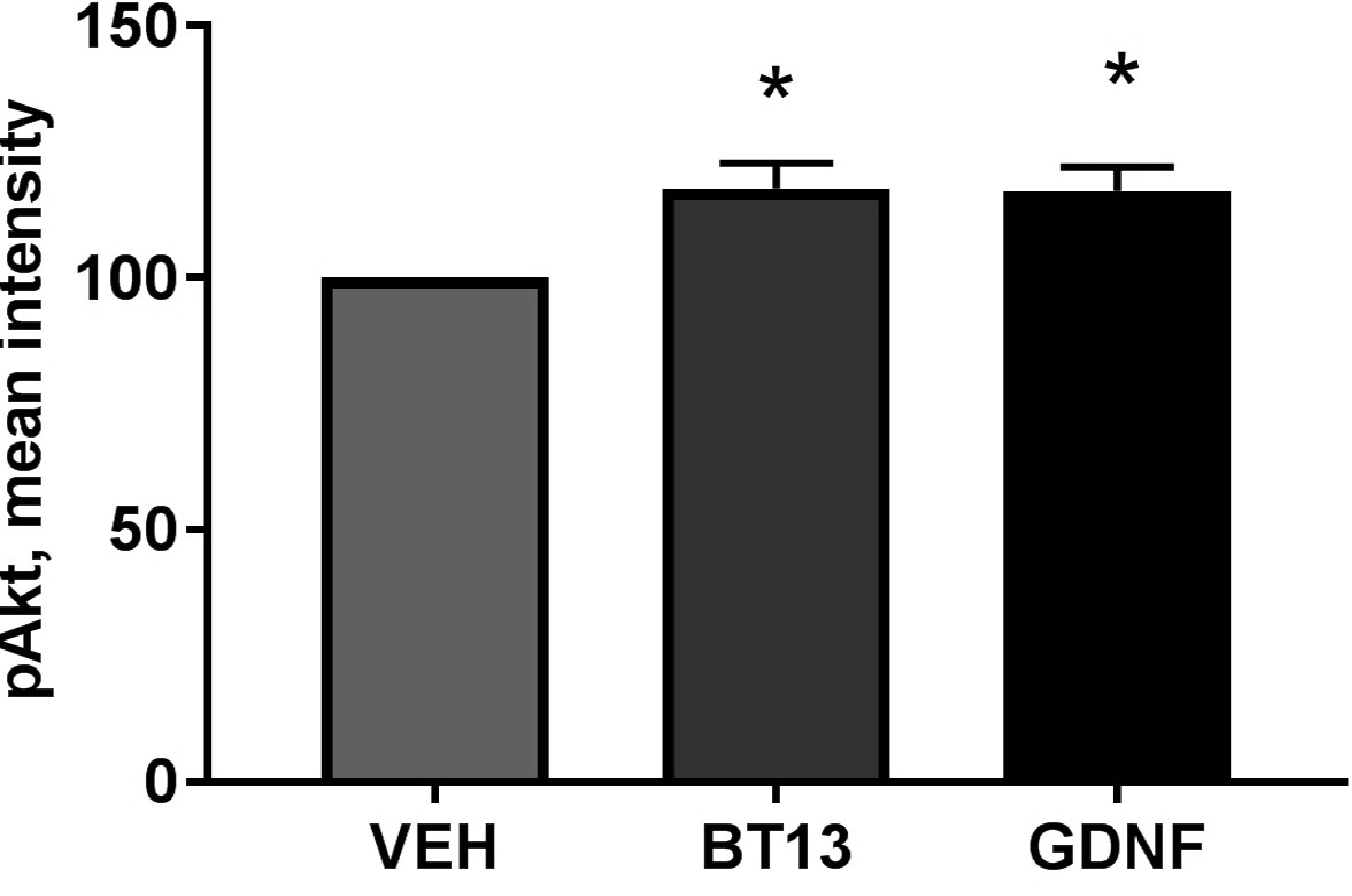
pAkt, mean intensity
150
100
50
0
*
*
VEH
BT13
GDNF

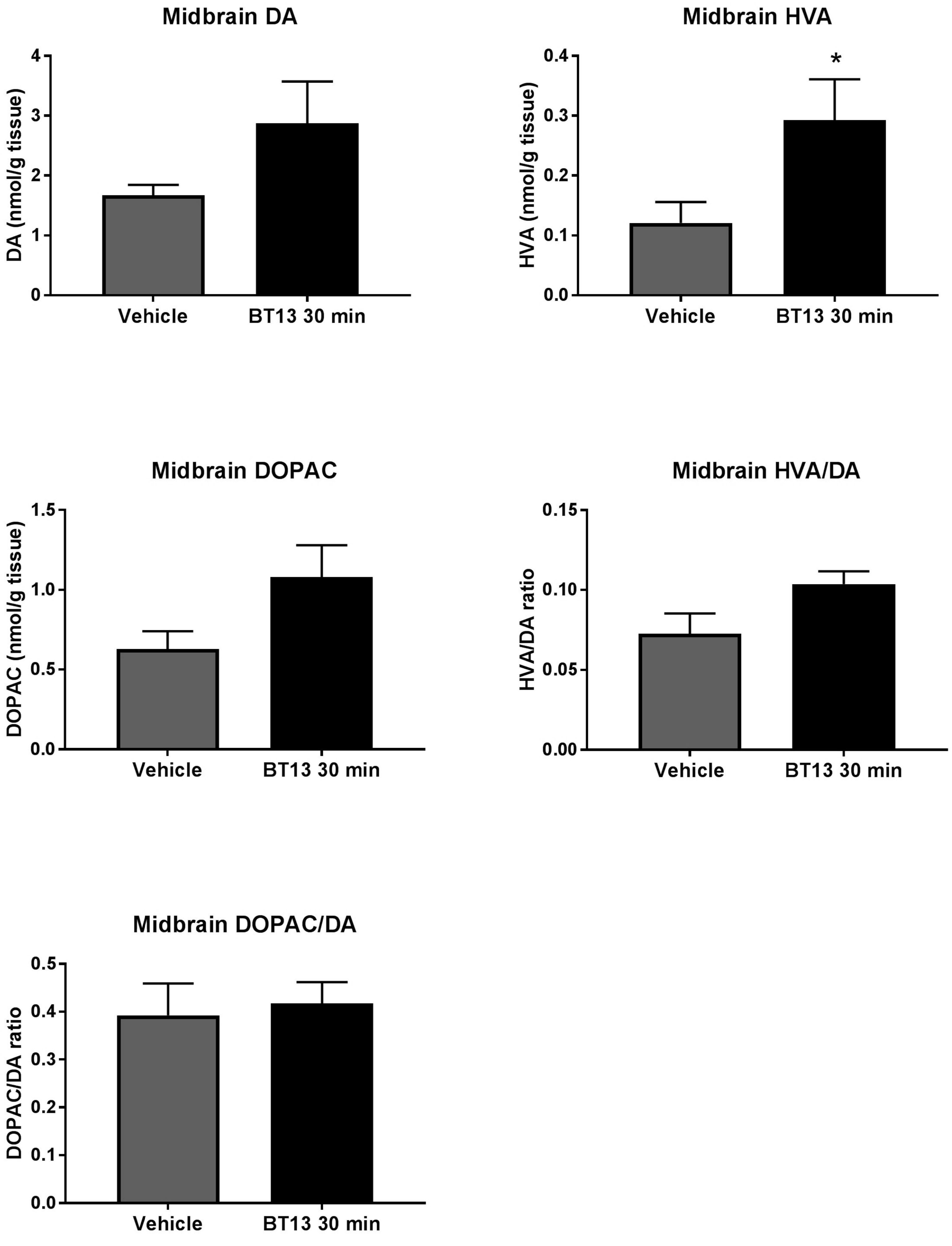

Midbrain DA
DA (nmol/g tissue)
0
1
2
3
4
Vehicle
BT13 30 min
Midbrain HVA
HVA (nmol/g tissue)
0.0
0.1
0.2
0.3
0.4
*
Vehicle
BT13 30 min
Midbrain DOPAC
DOPAC (nmol/g tissue)
0.0
0.5
1.0
1.5
Vehicle
BT13 30 min
Midbrain HVA/DA
HVA/DA ratio
0.00
0.05
0.10
0.15
Vehicle
BT13 30 min
Midbrain DOPAC/DA
DOPAC/DA ratio
0.0
0.1
0.2
0.3
0.4
0.5
Vehicle
BT13 30 min

Supplementary Table

Supplementary Table 1: Pharmacokinetics of BT13 and its brain distribution. Concentrations of BT13 in plasma, frontal cortex, striatum, midbrain, and cerebellum after intravenous injection of 10 mg/kg of compound. The concentrations in ng/ml or ng/g are presented as Mean ± SEM. Number of animals (N)=6 in each group

Time, Hours	Plasma, ng/ml	Frontal cortex, ng/g	Frontal Cortex, % from plasma concentration	Striatum, ng/g	Striatum, % from plasma concentration	Midbrain, ng/g	Midbrain, % from plasma concentration	Cerebellum	Cerebellum, % from plasma concentration
0.50	113.9 ± 16.84	301.5± 60.04	264.7	200.2 ± 39.36	175.8	277.6 ± 44.81	243.74	361.5 ± 85.58	317.4
1.00	75.1 ± 18.39	154.2± 20.98	205.3	131.9 ± 20.53	175.7	144.2 ± 19.82	192.03	178.1 ± 23.02	237.1
2.00	69.9 ± 6.10	100.4 ± 7.87	143.6	96.0 ± 8.21	137.3	89.07 ± 7.16	127.36	125.3 ± 8.75	179.1

References

1. Eketjäll S, Fainzilber M, Murray-Rust J, Ibáñez CF. Distinct structural elements in GDNF mediate binding to GFRα1 and activation of the GFRα1-c-Ret receptor complex. EMBO J. 1999;18(21):5901–10.

2. Sidorova YA, Mätlik K, Paveliev M, Lindahl M, Piranen E, Milbrandt J, et al. Persephin signaling through GFRα1: The potential for the treatment of Parkinson's disease. Mol Cell Neurosci. 2010;44(3):223–32.

3. Schuchardt A, D'Agati V, Pachnis V, Costantini F. Renal agenesis and hypodysplasia in ret-k- mutant mice result from defects in ureteric bud development. Development. 1996;122:1919–29.

4. Sidorova YA, Bespalov MM, Wong AW, Kambur O, Jokinen V, Lilius TO, et al. A Novel Small Molecule GDNF Receptor RET Agonist , BT13 , Promotes Neurite Growth from Sensory Neurons in Vitro and Attenuates Experimental Neuropathy in the Rat. Front Parmacology. 2017;8:1–18.

5. Leppanen VM, Bespalov MM, Runeberg-Roos P, Puurand U, Merits A, Saarma M, et al. The structure of GFRalpha1 domain 3 reveals new insights into GDNF binding and RET activation. EMBO J. 2004/03/27. 2004;23(7):1452–62.

6. Parkash V, Leppanen VM, Virtanen H, Jurvansuu JM, Bespalov MM, Sidorova YA, et al. The structure of the glial cell line-derived neurotrophic factor-coreceptor complex: insights into RET signaling and heparin binding. J Biol Chem. 2008;283(50):35164–72.

7. Planken A, Porokuokka LL, Hanninen AL, Tuominen RK, Andressoo JO. Medium-throughput computer aided micro-island method to assay embryonic dopaminergic neuron cultures in vitro. J Neurosci Methods. 2010;194(1):122–31.

8. Saarenpää T, Kogan K, Sidorova Y, Mahato AK, Tascón I, Kaljunen H, et al. Zebrafish GDNF and its co-receptor GFRα1 activate the human RET receptor and promote the survival of dopaminergic neurons in vitro. PLoS One. 2017;e0176166.

9. Carpenter AE, Jones TR, Lamprecht MR, Clarke C, Kang IH, Friman O, et al. CellProfiler: image analysis software for identifying and quantifying cell phenotypes. Genome Biol. 2006;7(10):R100.

10. Lotharius J, Dugan LL, O'Malley KL. Distinct Mechanisms Underlie Neurotoxin-Mediated Cell Death in Cultured Dopaminergic Neurons. J Neurosci. 1999;19(4):1284–93.

11. Schinelli S, Zuddas A, Kopin IJ, Barker JL, di Porzio U. 1-Methyl-4-Phenyl-1,2,3,6-Tetrahydropyridine Metabolism and l-Methyl-4-Phenylpyridinium Uptake in Dissociated Cell Cultures from the Embryonic Mesencephalon. J Neurochem. 1988;50(6):1900–7.

12. Hyman C, Hofer M, Barde Y, Juhasz M, Yancopoulos G, Squinto S, et al. BDNF is a neurotrophic factor for dopaminergic neurons of the substantia nigra. Nature. 1991;350:230–2.

13. Chmielarz P, Er Ş, Konovalova J, Bandrés L, Hlushchuk I, Albert K, et al. GDNF/RET signaling pathway activation eliminates Lewy Body pathology in midbrain dopamine neurons. BioRxiv. 2019;1–40.

14. Saarenpää T, Kogan K, Sidorova Y, Mahato AK, Tascón I, Kaljunen H, et al. Zebrafish GDNF and its co-receptor GFRα1 activate the human RET receptor and promote the survival of dopaminergic neurons in vitro. PLoS One. 2017;12(5):e0176166.

15. Paxinos G, Franklin KBJ. The mouse brain in stereotaxic coordinates. San Diego: Academic Press; 2001. 296 p.

16. Voutilainen MH, Back S, Porsti E, Toppinen L, Lindgren L, Lindholm P, et al. Mesencephalic astrocyte-derived neurotrophic factor is neurorestorative in rat model of Parkinson's disease. J Neurosci. 2009;29(30):9651–9.
